# Assessment of Radiographic Image Texture in the Maxilla and Mandible Around Titanium Inserts Used for Osteosynthesis of Dentofacial Deformities

**DOI:** 10.3390/jfb17010002

**Published:** 2025-12-19

**Authors:** Bożena Antonowicz, Marta Borowska, Kamila Łukaszuk, Łukasz Woźniak, Anna Zalewska, Alessia Distefano, Jan Borys

**Affiliations:** 1 Department of Dental Surgery, Medical University of Bialystok, 15-089 Bialystok, Poland; lukasz.wozniak@umb.edu.pl; 2 Institute of Biomedical Engineering, Faculty of Mechanical Engineering, Bialystok University of Technology, 15-351 Bialystok, Poland; 3 Department of Maxillofacial and Plastic Surgery, Medical University of Bialystok, 15-089 Bialystok, Poland; lukaszuk-kamila@wp.pl (K.Ł.); jan.borys@umb.edu.pl (J.B.); 4 Independent Laboratory of Experimental Dentistry, Medical University of Bialystok, 15-089 Bialystok, Poland; anna.zalewska1@umb.edu.pl; 5 Department of Restorative Dentistry, Medical University of Bialystok, 15-089 Bialystok, Poland; 6 Department of Chemical Sciences, University of Catania, 95125 Catania, Italy; alessiadistefano92@tiscali.it

**Keywords:** titanium fixations, jawbone fixations, miniplates and screws, titanium implants, texture analysis, radiological images

## Abstract

**Background**: In the treatment of dentofacial deformities, miniplates and screws made of titanium and its alloys (Ti6Al4V) are currently used for osteosynthesis of bone segments, which is due to the high biocompatibility of these materials. Despite the unquestionable advantages of titanium implants, there is an ongoing discussion about their potential negative impact on the human body, both at the implantation site and systemically. This study aimed to assess the influence of titanium fixations (miniplates and screws) on the texture and to identify the texture features that vary in the surrounding bone tissue. **Methods**: The orthopantomograms were obtained from 20 patients who were treated at the Department of Maxillofacial and Plastic Surgery, University of Bialystok. Regions of Interest (ROIs) of bone tissue surrounding titanium fixations in the maxilla and mandible were annotated using separate masks and compared to healthy areas of the same structures in the same patients. The images were independently filtered using Mean, Median, and Laplacian Sharpening filters, followed by analysis of the texture parameters obtained through methods such as First-Order Statistics (FOS), the Gray-Level Co-occurrence Matrix (GLCM), Neighbouring Gray Tone Difference Matrix (NGTDM), Gray-Level Dependence Matrix (GLDM), Gray-Level Run Length Matrix (GLRLM), and Gray-Level Size Zone Matrix (GLSZM). **Results**: The results showed that FOS, GLCM, and GLDM provide the most informative features for quantitative assessment of the areas around titanium fixations, and that smoothing filters reduce measurement noise and artifacts. **Conclusions**: The findings confirm that texture analysis can support the diagnosis of structural alterations in the bone surrounding titanium fixations, in both the maxilla and mandible.

## 1. Introduction

Titanium fixations in the form of miniplates and screws are widely used in maxillofacial surgery to immobilize jawbone fragments after trauma and osteotomies performed in the treatment of dentofacial deformities [1,2,3].

This is due to the high biocompatibility of titanium alloys, from which these fixations are made, as well as their significant corrosion resistance and favorable biomechanical properties. However, with the widespread use of titanium implants, there has been an increasing number of reports of adverse effects caused by their presence in the human body. These effects include complications in bone healing, inflammatory conditions, allergic reactions, and toxic responses, primarily at the site of titanium bone fixation implantation [4,5]. Around such implants, grayish discoloration of tissues has been observed, which may be caused by the release of titanium particles during the insertion of miniplates and screws, as well as by metallosis in their vicinity [6,7]. This results from the degradation of the metal or its alloys due to loss of structural integrity of the implant, caused by fretting and the associated electrochemical corrosion process (fretting corrosion) [8,9]. Fretting is a specific type of friction that occurs with minor micromovements in movable connections stabilizing segments of bone, leading to intensified tribological wear, fretting corrosion, and fatigue-related degradation of materials used in jawbone fixations [10].

Intense degradation processes, typical of corrosive and tribological damage such as abrasion, adhesion, and material fatigue, have been observed mainly on the contact surfaces of plate holes and the heads of bone screws. This has resulted in damage to the passive (protective) layer covering the implant surfaces [10,11,12,13]. In vitro studies have shown that fretting corrosion in fixation plates is the primary corrosion mechanism, initiated by micromovements at connection points, which intensify in the presence of bodily fluids [10,12]. The phenomenon of metallosis can be observed in tissues surrounding jawbone fixations during their removal. This has been demonstrated in both our research and studies by other authors, which have identified gray discoloration of the periosteum and bone, as well as excessive periosteal bone overgrowth at the site of miniplate and screw placement [10,14,15,16].

In our previous studies, we demonstrated alterations in oxidative stress parameters and redox balance in erythrocytes, blood serum, and the periosteum of patients with mandibular fractures and those undergoing surgery for maxillofacial deformities [14,17]. We assessed enzymatic and non-enzymatic antioxidant systems, as well as oxidative damage to proteins, lipids, and DNA in the periosteum surrounding bone fixations and in the plasma and erythrocytes of patients treated for mandibular fractures, compared with a control group [17,18,19]. In these patients, we also examined the impact of titanium bone fixations on glutathione metabolism, mitochondrial activity, ROS production, and selected markers of nitrosative and oxidative stress, as well as the concentration of pro-oxidative enzymes, cytokines, and pro- and anti-apoptotic proteins in the mandibular periosteum [15,16]. Additionally, in patients with mandibular fractures, after osteosynthesis using titanium miniplates and screws, we analyzed the effects of these fixations on antioxidant defense parameters, oxidative and nitrosative stress, and inflammation in plasma and erythrocytes [17].

We also evaluated the concentrations of cytokines, chemokines, growth factors, diacylglycerol, and sphingolipids released from the periosteum of the maxilla and mandible surrounding titanium fixations 11 months after the implantation procedure, both locally and in blood serum, in patients with dentofacial deformities [14,18,19].

All conducted studies revealed biochemical disturbances in the periosteum of the maxilla and mandible surrounding titanium bone fixations, as well as in blood serum [14,15,16,17].

We performed histopathological examinations of the removed periosteum, which revealed inflammatory cell infiltration [16].

We also conducted a chemical composition analysis of the mandibular periosteum (after osteosynthesis using titanium miniplates and screws) to detect metal particles using a Hitachi S-3000N (Hitachi High-Tech Corporation, Tokyo, Japan) scanning electron microscope equipped with an X-ray spectrometer. This analysis confirmed the presence of titanium, aluminum, and vanadium particles in the tissue [15]. Additionally, we assessed the condition of titanium miniplates before and after implantation, finding that the surface of the miniplate after removal was entirely different from that before implantation [16].

Data from the literature further indicate that titanium particles have been detected not only in tissues surrounding the implant but also in distant organs such as the lymph nodes, lungs, spleen, and liver [10,20]. It has been shown that the presence of a titanium implant and the metallic particles released from it can trigger an immune and inflammatory response to the foreign body. According to some authors, this may even contribute to carcinogenesis [21].

Previous studies have been conducted on generally healthy patients with uncomplicated bone healing. They evaluated various biochemical parameters in blood serum collected routinely before surgery, as well as in the periosteum covering titanium bone fixations, which is removed and discarded during implant removal. These studies are entirely safe and non-invasive for the patient. The structure of the bone tissue surrounding miniplates and screws has been assessed only macroscopically during surgery and on radiological examinations, which are subjective and may be inaccurate. A precise evaluation of the bone around titanium fixations seems highly relevant, but obtaining bone tissue samples for research from patients with asymptomatic healing would be highly invasive and clinically unjustified. Despite the numerous advantages of titanium bone fixations, the debate continues regarding their potential negative impact on the human body, both at the implantation site and systemically.

Texture analysis in diagnostic imaging provides a tool for extracting relevant features to assess bone structure in the mandible and maxilla by quantifying complexity and patterns in medical images [22,23,24]. Texture analysis enables the extraction of subtle information that may be imperceptible to the human eye but is crucial for distinguishing between different conditions. There are many mathematical methods of texture evaluation [25,26]. The most commonly used methods are based on creating matrices and accounting for the different behavior of pixel intensity occurrences in an image. The most important image analysis methods in this approach are as follows: First-Order Statistics (FOS) [26], the Gray-Level Co-occurrence Matrix (GLCM) [27], Neighbouring Gray Tone Difference Matrix (NGTDM) [28], Gray-Level Dependence Matrix (GLDM) [29], Gray-Level Run Length Matrix (GLRLM) [30,31], and Gray-Level Size Zone Matrix (GLSZM) [32].

Therefore, in this study, we analyzed bone in the vicinity of titanium fixations using mathematical methods to assess its texture compared to unchanged bone tissue.

## 2. Materials and Methods

### 2.1. Data Collection

The study included 20 patients (15 women and 5 men, aged 22–35 years, with a mean age of 25 years and 8 months) who were treated at the Department of Maxillofacial and Plastic Surgery, University of Bialystok, between 1 March 2024, and 30 April 2025, due to dentofacial deformities (Class II and III according to Angle’s classification—with growth disturbances of the maxilla and mandible affecting facial aesthetics and function). All patients underwent bilateral osteotomy of the maxilla and mandible. The osteotomized bone segments were stabilized using miniplates and screws made of Ti6Al4V alloy (ChM Lewickie, Sp. z o.o., Lewickie, Poland). Two miniplates, each fixed with four screws, were placed on the right and left sides of both the maxilla and the mandible. Titanium bone fixations were removed 11 months after the osteotomy, following the complete healing of the bone segments. Bone healing was assessed based on clinical examination, patient history, physical examination, intraoperative macroscopic evaluation, and radiological imaging—orthopantomogram (OPG). The orthopantomogram was performed using a panoramic and cephalometric extraoral dental radiograph (Orthopantomograph OP100 and Orthoceph OC100 dental X-ray system, Instrumentarium Imaging Inc., Jefferson City, MO, USA).

Only patients with a normal Body Mass Index (BMI) and no systemic diseases, such as cancer, autoimmune, metabolic, hematologic, psychiatric, rheumatologic, skeletal, cardiovascular, liver, or kidney diseases, as well as no eating disorders (anorexia, bulimia), were included in the study. They had normal biochemical test results within the reference ranges: WBC (4.00–10.00 × 10^3^/µL), RBC (4.50–6.00 × 10^6^/µL), HGB (14.0–18.0 g/dL), HCT (40.0–54.0%), MCV (80.0–94.0 fL), MCHC (31.0–37.0 g/dL), PLT (130–150 × 10^3^/µL), PT (12.0–16.0 s), APTT (26.0–40.0 s), INR (0.80–1.20), Na^+^ (136–145 mmol/L), K^+^ (3.5–5.1 mmol/L), Glucose (70.0–99.0 mg/dL), Creatinine (0.73–1.18 mg/dL), Urea (10.0–50.0 mg/dL), AST (5–34 U/I), ALT (0–55 U/I), CRP (0.0–10.0 mg/L). Patients did not take any medications, dietary supplements, or vitamins. They were not addicted to alcohol, tobacco, or drugs.

In the study group, none of the patients had previously undergone treatment for bone fractures or any osteotomy procedures. They had no history or current local oral diseases such as pulpitis, periodontitis, mucositis, or osteomyelitis. They had not used dental restorations or dental implants, nor had they previously undergone implantation of titanium bone implants, joint prostheses, or vascular clamps.

Patients with poor oral hygiene, e-cigarette smokers, and those with general or local complications in the bone healing process were excluded from the study.

Patients qualified for maxillary and mandibular osteotomy followed a balanced diet, designed and supervised by a dietitian, one month before surgery, immediately after the procedure, and throughout the period until the removal of the titanium bone fixations.

The osteotomy procedure, fixation of osteotomized bone segments using miniplates and screws, and their subsequent removal were all performed under general anesthesia by the same maxillofacial surgery specialist (J.B.) along with his team of assistants. Throughout the entire treatment period, patients remained under medical supervision to monitor both their general health and local condition.

### 2.2. Image Processing

The processing steps for analyzing the texture of orthopantomograms included several stages. Firstly, image acquisition was performed. Next, the Regions of Interest (ROIs) were annotated. Following that, the input images were filtered using three algorithms: Mean, Median, and Laplacian Sharpening.

**Figure 1:**
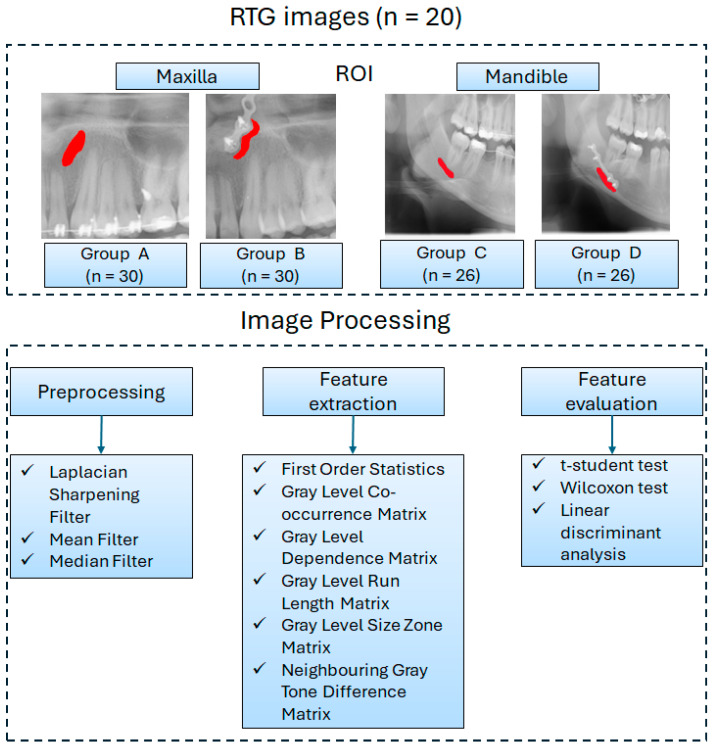
Scheme of image processing.

Then, texture features were extracted from the ROIs of the filtered images using six analytical methods: First-Order Statistics (FOS), the Gray-Level Co-occurrence Matrix (GLCM), Neighbouring Gray Tone Difference Matrix (NGTDM), Gray-Level Dependence Matrix (GLDM), Gray-Level Run Length Matrix (GLRLM), and Gray-Level Size Zone Matrix (GLSZM) (Figure 1). Each ROI was analyzed independently.

#### 2.2.1. Regions of Interest Annotation

The ROIs representing the bone tissue around the titanium fixations of the maxilla and mandible were manually annotated. Two experienced clinicians made the annotations. In the maxilla and mandible, the bone tissue area selected for examination on the OPG image was located near the outer edge of the miniplate on the right or left side, at the midpoint of its length, from the center to the lower pole (Figure 1). In the maxilla, the area of the maxillary sinus lumen was excluded, while in the mandible, the mandibular canal—through which nerves and blood vessels pass—was omitted. The patients were fitted with 1 to 4 miniplates.

The study results were compared with corresponding areas of healthy bone in both the maxilla and mandible on orthopantomograms of patients before undergoing osteotomy and the placement of titanium fixations. The masks were annotated using the ImageJ software (version 1.46r, Wayne Rasband, National Institutes of Health, Bethesda, MD, USA).

Based on the obtained results, the ROIs were assigned to the control group representing the healthy bone in both the maxilla (Group A, *n* = 30) and the mandible (Group C, *n* = 26), as well as to the study group located in the vicinity of the outer edge of the miniplates in both the maxilla (Group B, *n* = 30) and the mandible (Group D, *n* = 26).

#### 2.2.2. Filtering

In the second stage, radiographic images were filtered to minimize noise and improve image quality. Three specific methods were selected—mean filter, median filter, and Laplacian sharpening filter—due to their excellent performance in our previous image filtering studies [33]. These techniques showed the best results in improving the clarity and detail of radiographic images, making them the ideal choice for this analysis.

Mean filter is a commonly applied linear method in radiographic imaging that effectively smooths images by averaging pixel values [34,35]. Median filter is a widely used nonlinear technique for removing salt-and-pepper noise by identifying the median value within a local neighborhood around each pixel [35]. The Laplacian Sharpening filter is a powerful nonlinear method that enhances image sharpness by applying a Laplacian operator, which calculates the second derivative of the image [36].

The filtering algorithms were implemented in the SimpleITK module (https://simpleitk.org/, accessed on 15 July 2025, version 2.3.1) in Python version 3.11.4 [34,37,38].

#### 2.2.3. Image Texture Features

The following six texture analysis methods [26] were employed to extract 93 texture features from the radiographic images. These features were obtained from the segmented regions of interest (ROIs) of both the maxilla and mandible separately. The texture features were calculated independently for each filtered output image using PyRadiomics (https://pyradiomics.readthedocs.io/en/latest/index.html, accessed on 15 July 2025, version 3.0.1), an open-source Python package designed specifically for feature extraction from radiographic images [39].

The analysis of texture features employs a variety of statistical approaches, each contributing to a comprehensive understanding of pixel-intensity distributions in radiographic images.

Firstly, First-Order Statistics (FOS) use basic statistical parameters derived from the histogram, which serve as an empirical probability density function of the pixel intensities. Through this method, a total of 18 features were extracted [26]. Based on FOS, the Gray-Level Co-occurrence Matrix (GLCM) employs second-order statistics to calculate parameters from a matrix that represents the spatial interrelationships between pairs of pixels with specified intensity levels. This analysis accounts for different directions and distances between pixel pairs, resulting in 24 features [27]. Continuing with second-order statistics, the Gray-Level Dependence Matrix (GLDM) analyzes dependencies of gray levels within the image. It defines a gray-level dependency as the connectivity of pixels within a distance that is related to the central pixel. The elements of the GLDM represent the frequency with which a gray-level pixel depends on its surrounding pixels. This method returns 14 features [29]. Further expanding on the concept of gray levels, the Gray-Level Run Length Matrix (GLRLM) describes gray-level runs within the image, identifying consecutive pixels with the same gray-level value. The GLRLM approach returns 16 features [30,31]. Additionally, the Gray-Level Size Zone Matrix (GLSZM) examines gray-level zones within the image using second-order statistics. A gray-level zone is defined as a collection of connected pixels with identical gray-level intensity. Each element in the GLSZM signifies the frequency of zones defined by gray level and size. This method produces 16 features [40]. Lastly, the Neighbouring Gray Tone Difference Matrix (NGTDM) employs second-order statistics to derive parameters from the differences between a pixel’s gray level and the average gray value of its neighboring pixels at a Chebyshev distance. This matrix illustrates the sum of absolute differences in gray levels and consequently returns five features [28].

For each texture analysis method, the individual features derived from each matrix are comprehensively documented in the Supplementary Materials (Table S1).

### 2.3. Statistical Analysis

Data from 20 orthopantomographic images included separately annotated regions of interest (ROIs) representing healthy bone in both the maxilla (Group A, *n* = 30) and the mandible (Group C, *n* = 26), as well as in the study group located near the outer edge of the miniplates in both the maxilla (Group B, *n* = 30) and the mandible (Group D, *n* = 26). It is worth noting that the ROIs within the maxilla for both groups corresponded to the same anatomical structures, as did those within the mandible. Each ROI was characterized by texture parameters as detailed in Section 2.2.3.

Data were presented as individual series for each filtration, with each ROI representing one realization. Univariate distributions of the data series were independently tested for normality using the Shapiro–Wilk test. The paired *t*-test (for Gaussian distributed data) or the Wilcoxon signed-rank test (for non-Gaussian distributed data) was employed to compare the ROIs obtained before and after osteotomy of the maxilla and mandible.

The integrity of the statistical analysis and the risk of false-positive results were enhanced by applying a Bonferroni correction. This correction allows for control of the probability of misidentifying parameters as statistically significant. Further analysis focused only on statistically significant parameters (*p* < 0.05) and demonstrated statistical power of the test greater than 80%.

The significant features showing differences between the groups of the maxilla and mandible were reported as mean ± standard deviation (SD) and *p*-value, with additional details provided in the Supplementary Materials (Tables S2 and S3).

All statistical analyses were conducted using SciPy (https://scipy.org/, accessed on 15 July 2025, version 1.12.0) in Python version 3.11.4, with a significance level set at *p* < 0.05. The utility of these significant features was further evaluated using linear discriminant analysis (LDA). LDA is a linear supervised machine learning technique that facilitates the differentiation between two or more groups, providing optimal class separation by transforming features from a multidimensional space to a lower-dimensional space while maximizing the ratio of between-class variability to within-class variance [41]. In this study, LDA was also used as an additional feature selection technique in comparison to feature selection based on statistical analysis.

## 3. Results

A comprehensive analysis was conducted on 279 combinations of filtration methods (*n* = 3) and image texture features (*n* = 93). Each filtration method returned statistically significant texture characteristics, which were examined in both the maxilla and mandible.

**Figure 2:**
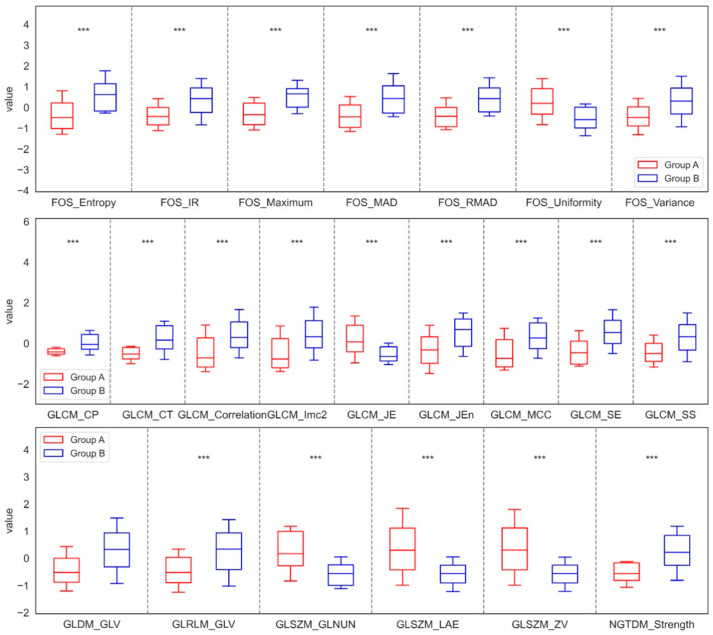
Features of First-Order Statistics (FOS), the Gray-Level Run Length Matrix (GLRLM), Neighbouring Gray Tone Difference Matrix (NGTDM), Gray-Level Dependence Matrix (GLDM), Gray-Level Size Zone Matrix (GLSZM), and Gray-Level Co-occurrence Matrix (GLCM), extracted from examined output images, filtered by the Laplacian Sharpening filter, compared between the control group (Group A) and the study group (Group B) of the maxilla. Differences between groups were indicated with individual *p*-values when *p* < 0.05 (*** *p* < 0.001).

**Figure 3:**
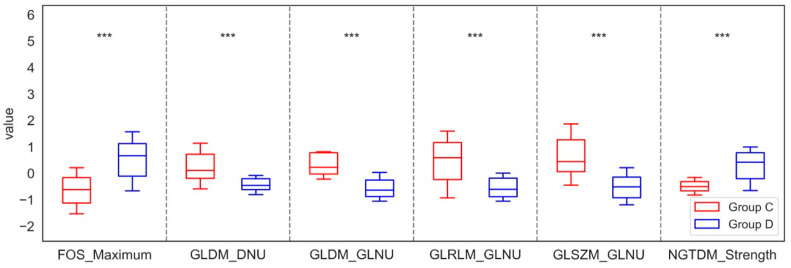
Features of First-Order Statistics (FOS), the Gray-Level Run Length Matrix (GLRLM), Neighbouring Gray Tone Difference Matrix (NGTDM), Gray-Level Dependence Matrix (GLDM), Gray-Level Size Zone Matrix (GLSZM), and Gray-Level Co-occurrence Matrix (GLCM), extracted from examined output images, filtered by the Laplacian Sharpening filter, compared between the control group (Group C) and the study group (Group D) of the mandible. Differences between groups were indicated with individual *p*-values when *p* < 0.05 (*** *p* < 0.001).

For the Laplacian sharpening filtration method, it was observed that in the maxilla, at least seven features from FOS, nine features from GLCM, one feature from GLDM, one feature from GLRLM, three features from GLSZM, and one feature from NGTDM showed significant differences between the control group (Group A) and the study group (Group B). Specifically, the results indicated that the values of Entropy, IR, Maximum, MAD, Variance, CP, CT, Correlation, Imc2, Jen, MCC, SE, SS, GLV, and Strength increased significantly in Group B compared to Group A. In contrast, the values of Uniformity, JE, GLNUN, LAE, and ZV decreased significantly in Group B compared to Group A. This suggests changes in bone density or microstructure. A more pronounced structure may indicate mineralization or bone remodeling, while variation in structure may be connected with abnormal changes. Conversely, for the mandible, significant differences were also identified, with at least one feature from FOS, two from GLDM, one from GLRLM, one from GLSZM, and one from NGTDM distinguishing the control group (Group C) from the study group (Group D). In this case, the analysis showed that the values of Entropy, IR, Maximum, MAD, RMAD, and Variance increased in Group D, whereas the value of Uniformity decreased relative to Group C. An increase in bone density may suggest an improvement in bone mineralization, which may be the result of increased bone remodeling. On the other hand, an increase in variance may indicate abnormal changes, possibly due to titanium implants and an irregular mineral distribution. These significant differences in the parameters of the LS filtration method of the maxilla and mandible were shown in Figure 2 and Figure 3.

**Figure 4:**
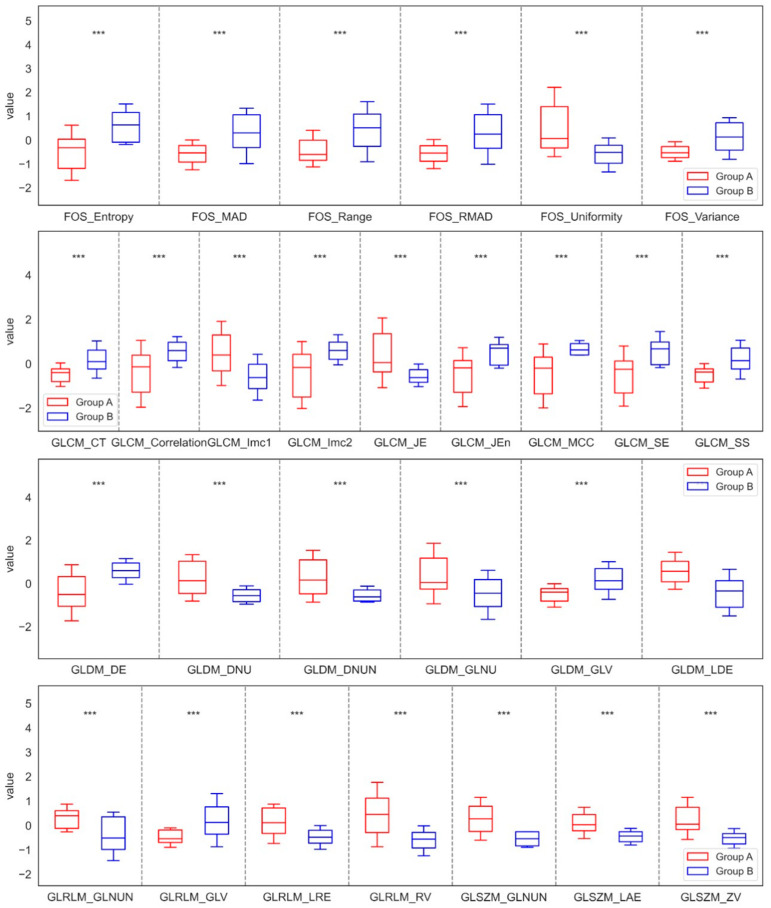
Features of First-Order Statistics (FOS), the Gray-Level Run Length Matrix (GLRLM), Neighbouring Gray Tone Difference Matrix (NGTDM), Gray-Level Dependence Matrix (GLDM), Gray-Level Size Zone Matrix (GLSZM), and Gray-Level Co-occurrence Matrix (GLCM), extracted from examined output images, filtered by a Mean filter compared between the control group (Group A) and the study group (Group B) of the maxilla. Differences between groups were indicated with individual *p*-values when *p* < 0.05 (*** *p* < 0.001).

**Figure 5:**
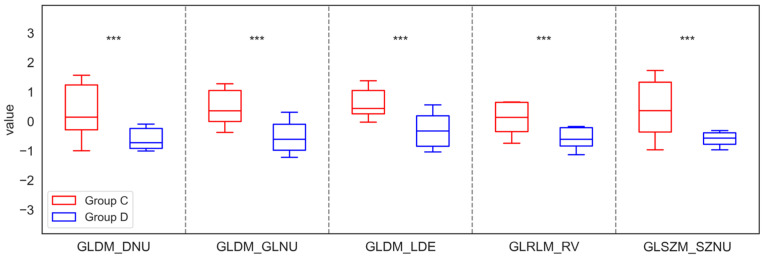
Features of First-Order Statistics (FOS), the Gray-Level Run Length Matrix (GLRLM), Neighbouring Gray Tone Difference Matrix (NGTDM), Gray-Level Dependence Matrix (GLDM), Gray-Level Size Zone Matrix (GLSZM), and Gray-Level Co-occurrence Matrix (GLCM), extracted from examined output images, filtered by a Mean filter, compared between the control group (Group C) and the study group (Group D) of the mandible. Differences between groups were indicated with individual *p*-values when *p* < 0.05 (*** *p* < 0.001).

After applying the Mean filter, the analysis also showed varied results, but very similar to those obtained with LS filtering. Within the maxilla, seven features of FOS, nine of GLCM, six of GLDM, four of GLRLM, and three of GLSZM showed significant differences between Groups A and B. The findings confirmed that the values of Entropy, Mad, Range, RMAD, Variance, CT, Correlation, Imc2, Jen, MCC, SE, SS, DE, and GLV increased in Group B compared to Group A. In contrast, the values of Uniformity, Imc1, JE, DNU, DNUN, GLNU, LDE, GLNUN, LRE, RV, GLNUN, LAE, and ZV decreased in Group B compared to Group A. For the mandible, significant differences were noted, characterized by three features from GLDM, one from GLRLM, and one from GLSZM. In this case, the results revealed that the values of DNU, GLNU, LDE, RV, and SZNU decreased in Group D compared with Group C. These significant differences in the parameters of the Mean filtration method for the maxilla and mandible are shown in Figure 4 and Figure 5.

**Figure 6:**
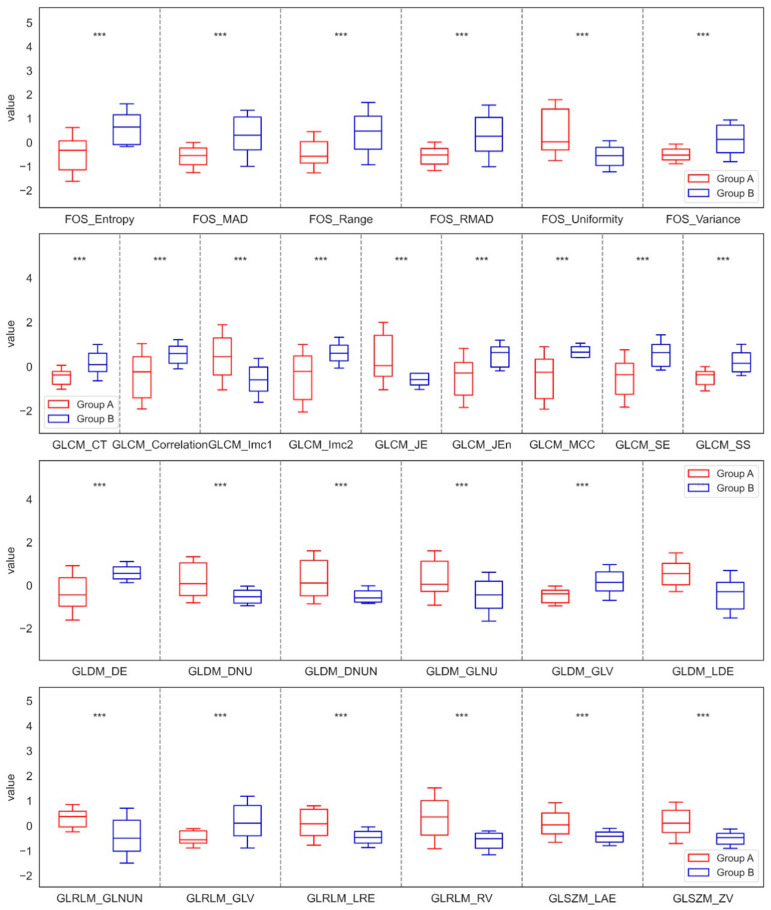
Features of First-Order Statistics (FOS), the Gray-Level Run Length Matrix (GLRLM), Neighbouring Gray Tone Difference Matrix (NGTDM), Gray-Level Dependence Matrix (GLDM), Gray-Level Size Zone Matrix (GLSZM), and Gray-Level Co-occurrence Matrix (GLCM), extracted from examined output images, filtered by a Median filter, compared between the control group (Group A) and the study group (Group B) of the maxilla. Differences between groups were indicated with individual *p*-values when *p* < 0.05 (*** *p* < 0.001).

**Figure 7:**
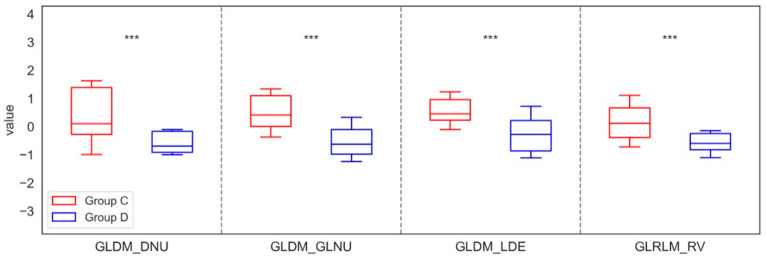
Features of First-Order Statistics (FOS), the Gray-Level Run Length Matrix (GLRLM), Neighbouring Gray Tone Difference Matrix (NGTDM), Gray-Level Dependence Matrix (GLDM), Gray-Level Size Zone Matrix (GLSZM), and Gray-Level Co-occurrence Matrix (GLCM), extracted from examined output images, filtered by a Median filter, compared between the control group (Group C) and the study group (Group D) of the mandible. Differences between groups were indicated with individual *p*-values when *p* < 0.05 (*** *p* < 0.001).

After median filtering in the maxilla, similar significant differences in parameters were found as for LS and Mean filters, specifically in six features for FOS, nine for GLCM, six for GLDM, four for GLRLM, and two for GLSZM when comparing group A with group B. Notably, the findings indicated that the values of Entropy, MAD, Range, RMAD, Variance, CT, Correlation, Imc2, Jen, MCC, SE, SS, DE, and GLV increased significantly in Group B compared to Group A, while the values of Uniformity, Imc1, JE, DNU, DNUN, GLNU, LDE, GLNUN, LRE, RV, LAE, and ZV decreased, highlighting the clinical importance of these texture changes. Similarly, significant differences in the mandible were observed only for three from GLDM and one from GLRLM, distinguishing Groups C and D. In this case, the analysis revealed that the values of DNU, GLNU, LDE, and RV decreased in Group D compared to Group C. These significant differences in the parameters of the Median filtration method for the maxilla and mandible are shown in Figure 6 and Figure 7.

The mean values and standard deviations for the significant parameters from both the maxilla (Groups A and B) and the mandible (Groups C and D) of LS, Mean, and Median filtration are presented in Supplementary Tables S2 and S3.

**Figure 8:**
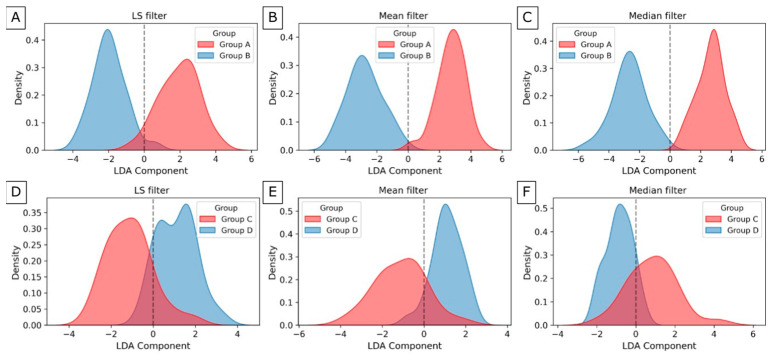
Density distribution of significance features for Groups A and B of the maxilla after Laplacian sharpening filter (**A**), Mean filter (**B**), Median filter (**C**), and for Groups C and D of the mandible after Laplacian sharpening filter (**D**), Mean filter (**E**), and Median filter (**F**).

The classification and dimensionality reduction technique, linear discriminant analysis (LDA), was applied. This technique finds a linear combination of features that best separates two or more classes by maximizing the distance between class means while minimizing variance within each class. From the significant texture dataset, 22 texture features of LS filtration, 28 of Mean filtration, and 27 of Median filtration were selected for maxilla group separation (Figure 8A–C). The plots overlap minimally around zero. However, the groups were separated along this component, and the distribution centers’ positions relative to the zero axis are on both sides of zero, with similar distributions. From the significant texture dataset, 23 texture features of LS filtration, 28 of mean filtration, and 23 of median filtration were selected for maxilla group separation (Figure 8D–F). The plots overlap around zero, while the distribution maxima are separated. Although the peaks of the distributions are separated, the presence of overlapping points indicates a significant overlap between the groups.

**Figure 9:**
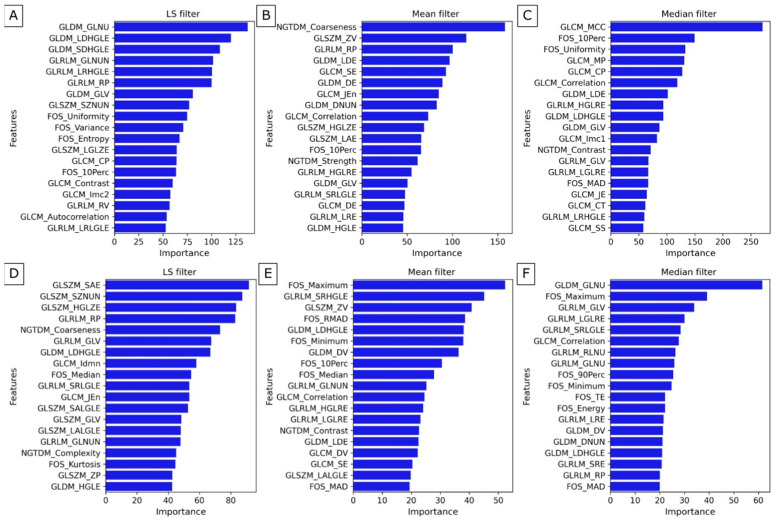
The importance of significant texture features of the maxilla dataset of (**A**) the Laplacian Sharpening filter, (**B**) the mean filter, and (**C**) the median filter, and of the mandible dataset of (**D**) the Laplacian Sharpening filter, (**E**) the mean filter, and (**F**) the median filter.

**Figure 10:**
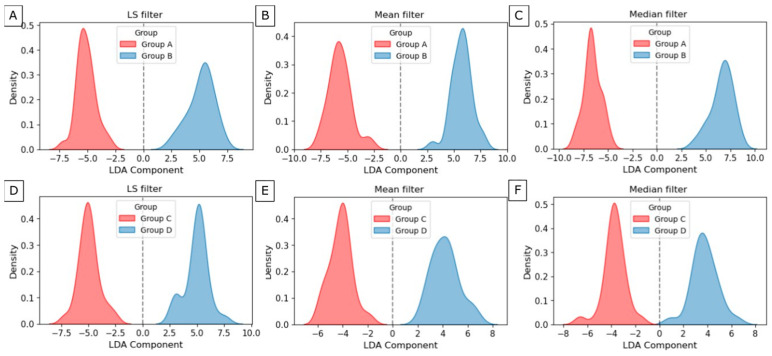
Density distribution for Groups A and B of the maxilla after Laplacian sharpening filter (**A**), Mean filter (**B**), Median filter (**C**), and for Groups C and D of the mandible after Laplacian sharpening filter (**D**), Mean filter (**E**), and Median filter (**F**).

To identify the features that contribute most to class separation, perform feature selection, and improve understanding of the underlying data structure, LDA was used. By projecting multidimensional data onto a lower-dimensional space, LDA simplifies the data while preserving the features most useful for classification. This is particularly useful for reducing computational complexity and improving model performance. Of all the texture features obtained after LS filtering of the maxilla, the most important are GLNU, LDHGLE, and SDHGLE from GLDM, and GLNUN, LRHGLE, and RP from GLRLM. Selecting important features describes the variability of gray levels, the distribution of bright pixels, the intensity of nearby dense fragments, and the presence of long strings of high-intensity pixels. These factors can help identify differences in bone tissue structure and biomechanical properties, which are crucial for evaluating the effectiveness of surgical interventions. In the case of the Mean filter, the most important features are Coarseness from NGTDM, ZV from GLSZM, RP from GLRLM, and LDE, and DE from GLDM, while in the case of the median filter, they are MCC, MP, CP, and Correlation from GLCM, and 10Perc, and Uniformity from FOS. In the case of the Mean filter, identified features describe the texture roughness, the size, and distribution of homogeneous regions, and the gray-level dependencies and their variations. In the case of the Median filter, selected features provide insights into the texture and arrangement of the bone tissue. Additionally, they can help in assessing the distribution and consistency of pixel values. The importance of these features is shown in Figure 9A–C. The visualization of group separation between A and B of the maxilla is shown in Figure 10A–C. The groups were separated along this component, and the distribution centers’ positions relative to the zero axis are on both sides of zero, with similar distributions.

Of all the texture features obtained after LS filtering of the mandible, the most important are SAE, SZNUN, and HGLZE from GLSZM, RP, and GLV from GLRLM, and Coarseness from NGTDM. In the case of the Mean filter, the most important features are common Maximum, RMAD, Minimum from FOS, and SRHGLE from GLRLM, and ZV from GLSZM, while in the case of the Median filter, they are GLNU from GLDM, and Maximum from FOS, and GLV, LGLRE, and SRLGLE from GLRLM. After LS and Mean filtrations, important descriptors provide valuable insights into the size and distribution of homogeneous regions, indicate the percentage of long runs and texture variation, and highlight the roughness of the bone tissue. After Median filtration, selected features measure the non-uniformity of gray levels in the bone structure, provide insights into the highest-intensity values, and emphasize variations in gray-level runs and texture continuity. The importance of these features is shown in Figure 9D–F. The visualization of group separation between C and D of the mandible is shown in Figure 10D–F. The groups were also separated along this component, and the distribution centers’ positions relative to the zero axis are on both sides of zero, with similar distributions.

## 4. Discussion

The initial cross-sectional studies of bone in the region of titanium fixations revealed differences in texture assessment parameters compared to the surrounding healthy bone tissue. Ninety-three texture parameters based on the first-order statistic (FOS) and the second-order statistic (GLCM, NGTDM, GLDM, GLRLM, and GLSZM) were tested, and the study indicated that most parameters are sensitive to changes in the area of titanium fixations. The results are based on 30 regions of the maxilla and 26 of the mandible for three filters: Laplacian Sharpening, Mean, and Median. The presented studies enabled the extraction of significant parameters with potential impact on the assessment of bone tissue around titanium fixations.

Titanium fixations (miniplates and screws) are used not only to immobilize jawbone fragments after fractures but also to stabilize bone segments following jaw osteotomies in patients with dentofacial deformities [16]. The presence of such artificial materials in the human body can result in complications in bone healing, inflammatory conditions, allergic reactions, and toxic responses [4,5]. Consequently, macroscopic, intraoperative, and radiological examinations of the bone tissue structure surrounding the miniplates and screws are conducted, although these methods are often highly subjective [10,14,15,16]. Therefore, an effort has been made to accurately evaluate bone tissue in the vicinity of titanium fixations using mathematical texture analysis, compared with surrounding healthy bone tissue.

Healthy maxillary bone consists of trabecular and cortical bone, with trabecular bone predominating. Trabecular bone is more porous and contains cavities filled with marrow. In contrast, healthy mandibular bone has a higher proportion of cortical bone, with smaller trabecular spaces that limit the amount of bone marrow. Consequently, the maxilla shows a lower density than the mandible. The structural differences between the maxillary and mandibular bones are significant [42,43,44]. However, both healthy maxillary and mandibular bones demonstrate specific structural properties that can be characterized through texture analysis, which uses mathematical methods to describe relationships among pixels of different gray levels. Such methods include First-Order Statistics (FOS), Gray-Level Co-occurrence Matrix (GLCM), Gray-Level Size Zone Matrix (GLSZM), and Neighbouring Gray Tone Difference Matrix (NGTDM). Texture features can vary widely depending on image characteristics, preprocessing techniques, and the parameters used in the applied mathematical methods [33,45,46].

A review of the literature shows that few studies have examined texture analysis in this context, so it is important to define texture parameters that can demonstrate changes in the microstructure of healthy maxillary and mandibular bones relative to the surrounding tissue [22,23,24]. However, research on changes in the region of titanium fixations and their influence on the structure is insufficient, particularly in terms of texture analysis.

Our study, for the maxilla, showed significant differences between Group A and Group B in both first-order statistics (FOS) and second-order statistics (GLCM, GLRLM, GLDM, GLSZM, and NGTDM), regardless of the applied filter: Laplacian Sharpening, Mean, or Median. All applied filters were discriminative, whereas the Mean filter was the most. For FOS, there was an increase in Entropy, MAD, Range, RMAD, and Variance, along with a decrease in Uniformity around the titanium fixations. These changes indicate greater variability in pixel intensity, disorganization of pixel arrangement, or reduced homogeneity compared to standard bone structure. Regarding GLCM, which describes spatial relationships between pixels with different intensity levels, features such as CT, Correlation, Imc2, JEn, MCC, SE, and SS increased in the vicinity of titanium fixations, while Imc1 and JE decreased, regardless of the filter used. These differences suggest asymmetry in pixel distribution, greater variability in pixel intensity, greater heterogeneity and disorganization in the structural pattern, and a loss of the dominant pattern characteristic of trabecular bone and marrow spaces. For the GLRLM, GLDM, and GLSZM matrices, similar features such as GLNU, GLNUN, and GLV are observed. GLNU and GLNUN decreased significantly in Group B, while GLV increased regardless of the filter applied, indicating greater intensity heterogeneity (GLV) and the presence of homogeneous regions or zones relative to the more porous, marrow-filled structure typical of normal bone (reflected by GLNU and GLNUN). Analysis of the GLRLM also showed that LRE and RV decreased significantly in Group B, suggesting the emergence of additional regions with different structures than normal. In the case of GLSZM, features such as LAE and ZV decreased significantly in Group B, indicating the appearance of additional homogeneous zones. Regarding NGTDM, Strength exhibited higher values near the titanium fixations regardless of the filtering method, making these parameters the most discriminative between the studied groups. This suggests that the structure in Group B is more complex, with greater irregularities in pixel intensity and the presence of patterns that deviate from the standard structure.

In the mandible, fewer features from both first-order statistics (FOS) and second-order statistics (GLCM, GLRLM, GLDM, GLSZM, and NGTDM) were identified to differentiate between standard bone structure and the area around titanium fixations. Similar to the maxilla, the most significant differences were observed with the Mean filter, while the least were noted with the Laplacian Sharpening filter. For FOS, a significant increase in Maximum was observed in Group D, indicating the presence of bright pixels, resulting in a less homogeneous texture. In the case of GLDM, a decrease in DNU and GLNU values was observed around the titanium fixations regardless of the applied filtering, potentially indicating the development of other homogeneous structures. Similar changes in GLNU features were observed in GLRLM and GLSZM. In the NGTDM, after applying Laplacian Sharpening the most significant differences between the studied groups were observed. Strength significantly increased around the titanium fixations. Homogeneous structures make it difficult to detect subtle changes during bone remodeling, as evidenced by slight variations in texture parameters.

The obtained results—changes in texture measures—may reflect modifications in the structure of bone trabeculae and the marrow space surrounding titanium fixations in both the maxilla and the mandible. These phenomena may be associated with the healing and remodeling of bone tissue following jaw osteotomy, as well as with titanium fixation subjected to masticatory forces. These loads, transmitted to the osteotomized segments through the stabilizing miniplates and screws, may induce stress in the surrounding bone, which in turn may alter its architecture.

Previous reports have described phenomena such as tribocorrosion, oxidative stress, and disturbances in lipid metabolism, all of which may also influence bone remodeling in the vicinity of titanium fixations.

It should be emphasized that despite the observed differences between the examined bone regions and healthy tissue, no clinical disturbances in healing or signs of inflammation were noted in the vicinity of the titanium miniplates and screws. However, their long-term impact on bone tissue metabolism cannot be excluded.

The conducted linear discriminant analysis revealed which features enable separation between groups in the maxilla, with tissue structures showing apparent differences. Fewer discriminative features were identified in the mandible, and the LDA showed overlapping groups, which may result from structural differences between the bone tissue of the maxilla and the mandible. The results also showed that the method used to select important features is crucial for building a diagnostic model.

Considering the reproducibility of the features across different approaches and their specific pattern, it can be concluded that the selection of filtering algorithms, texture analysis methods, and selecting features for evaluating the area around titanium fixations should include the analysis of FOS, GLCM, and GLDM features from the output images obtained after filtering with the Mean filter. However, further research is necessary to assess the utility of texture analysis of radiographic images around miniplates, including the examination of changes over a longer time interval.

A limitation of the study is the difficulty in interpreting the obtained bone tissue texture parameters, due to the limited number of publications on this topic and the lack of studies specifically addressing the bones of the facial skeleton. Additionally, orthopantomograms have limitations that affect diagnostic accuracy and the assessment of bone structure. One of the main limitations is the low image resolution, which makes it challenging to visualize small pathological changes or subtle differences in bone structure. Furthermore, OPT provides only a two-dimensional view.

A potential direction for future research may be to evaluate bone tissue texture in both normal regenerative processes and inflammation-related complications using CBCT (Cone Beam Computed Tomography), which provides three-dimensional images with significantly higher resolution.

## 5. Conclusions

Texture analysis of radiographic images, using various filters and feature-extraction methods based on first- and second-order statistics, enables the identification of significant differences in the bone tissue surrounding titanium fixations in the maxilla and mandible compared with healthy bone. In the maxilla, GLCM parameters provide the most valuable information for the quantitative assessment of bone tissue, reflecting changes in pixel-intensity distribution, heterogeneity, and texture asymmetry. Typically, increases in features such as Cluster Tendency and Sum Entropy indicate higher heterogeneity and disorganization of the tissue structure. Conversely, indicators such as Joint Energy, Maximum Probability, GLNU, and GLNUN decrease relative to those in healthy bone, suggesting the emergence of more diverse and less organized structural patterns. In the mandible, fewer variables and minor differences between groups were observed. However, strength increased, reflecting changes in the distribution of texture. Additionally, filtering enhances the utility of extracted features as markers for differentiating between bone changes. The findings confirm that texture analysis can support the diagnosis of structural alterations in the bone surrounding titanium fixations, in both the maxilla and mandible.

## Data Availability

The data presented in this study are available on request from the corresponding author. The data are not publicly available due to ethical restrictions.

## Supplementary Materials

The following are available online at https://www.mdpi.com/article/10.3390/jfb17010002/s1. Table S1: Texture analysis approaches and their features, Table S2: The values of features (mean ± SD) of First-Order Statistics (FOS), Gray-Level Co-occurrence Matrix (GLCM), Gray-Level Size Zone Matrix (GLSZM), and Neighbouring Gray Tone Difference Matrix (NGTDM) of output images, filtrated by Laplacian Sharpening, Mean, and Median filter between Group A and Group B of the maxilla, Table S3: The values of features (mean ± SD) of First-Order Statistics (FOS), Gray-Level Co-occurrence Matrix (GLCM), Gray-Level Size Zone Matrix (GLSZM), and Neighbouring Gray Tone Difference Matrix (NGTDM) of output images, filtrated by Laplacian Sharpening, Mean, and Median filter between Group C and Group D of the mandible.

## Abbreviations

The following abbreviations are used in this manuscript:

ROI	Region of Interest
FOS	First-Order Statistics
GLCM	Gray-Level Co-occurrence Matrix
NGTDM	Neighbouring Gray Tone Difference Matrix
GLDM	Gray-Level Dependence Matrix
GLRLM	Gray-Level Run Length Matrix
GLSZM	Gray-Level Size Zone Matrix
10Perc	10th Percentile
90Perc	90th Percentile
RMS	Root Mean Squared
IR	Interquartile Range
MAD	Mean Absolute Deviation
RMAD	Robust Mean Absolute Deviation
TE	Total Energy
CP	Cluster Prominence
CS	Cluster Shade
CT	Cluster Tendency
DA	Difference Average
DE	Difference Entropy
DV	Difference Variance
Id	Inverse Difference
Idm	Inverse Difference Moment
Idmn	Inverse Difference Moment Normalized
Idn	Inverse Difference Normalized
Imc 1	Informational Measure of Correlation 1
Imc 2	Informational Measure of Correlation 2
MCC	Maximal Correlation Coefficient
MP	Maximum Probability
SA	Sum Average
SE	Sum Entropy
SS	Sum of Squares
DEn	Dependence Entropy
DN	Dependence Non-uniformity
DNN	Dependence Non-Uniformity Normalized
DV	Dependence Variance
GLN	Gray-Level Non-uniformity
GLNN	Gray-Level Non-uniformity Normalized
GLV	Gray-Level Variance
HGLE	High Gray-Level Emphasis
LDE	Large Dependence Emphasis
LDHGLE	Large Dependence High Gray-Level Emphasis
LDLGLE	Large Dependence Low Gray-Level Emphasis
LGLE	Low Gray-Level Emphasis
SDE	Small Dependence Emphasis
SDHGLE	Small Dependence High Gray-Level Emphasis
SDLGLE	Small Dependence Low Gray-Level Emphasis
HGLRE	High Gray-Level Run Emphasis
LRE	Long Run Emphasis
LRHGLE	Long Run High Gray-Level Emphasis
LRLGLE	Long Run Low Gray-Level Emphasis
LGLRE	Low Gray-Level Run Emphasis
RE	Run Entropy
RLN	Run Length Non-Uniformity
RLNN	Run Length Non-Uniformity Normalized
RP	Run Percentage
RV	Run Variance
SRE	Short Run Emphasis
SRHGLE	Short Run High Gray-Level Emphasis
SRLGLE	Short Run Low Gray-Level Emphasis
HGLZE	High Gray-Level Zone Emphasis
LAE	Large Area Emphasis
LAHGLE	Large Area High Gray-Level Emphasis
LALGLE	Large Area Low Gray-Level Emphasis
LGLZE	Low Gray-Level Zone Emphasis
SZN	Size-Zone Non-Uniformity
SZNN	Size-Zone Non-Uniformity Normalized
SAE	Small Area Emphasis
SAHGLE	Small Area High Gray-Level Emphasis
SALGLE	Small Area Low Gray-Level Emphasis
ZE	Zone Entropy
ZP	Zone Percentage
ZV	Zone Variance
