# Supplementary material for: Assessment of Radiographic Image Texture in the Maxilla and Mandible Around Titanium Inserts Used for Osteosynthesis of Dentofacial Deformities
Source: J Funct Biomater. 2025 Dec 19;17(1):2. doi: 10.3390/jfb17010002 (PMC12842460; doi:10.3390/jfb17010002)
Supplement: Supplementary file 1 [file jfb-17-00002-s001.zip › jfb-4015905-supplementary.pdf]

Supplementary Data for

Assessment of Radiographic Image Texture in the Maxilla and Mandible Around Titanium Inserts Used for Osteosynthesis of Dentofacial Deformities

Bożena Antonowicz [1,*], Marta Borowska [2,*], Kamila Łukaszuk [3], Łukasz Woźniak [1], Anna Zalewska [4,5], Alessia Distefano [6] and Jan Borys [3]

[1] Department of Dental Surgery, Medical University of Bialystok, 15-089 Bialystok, Poland; lukasz.wozniak@umb.edu.pl
[2] Institute of Biomedical Engineering, Faculty of Mechanical Engineering, Bialystok University of Technology, 15-351 Bialystok, Poland
[3] Department of Maxillofacial and Plastic Surgery, Medical University of Bialystok, 15-089 Bialystok, Poland; lukaszuk-kamila@wp.pl (K.Ł.); jan.borys@umb.edu.pl (J.B.)
[4] Independent Laboratory of Experimental Dentistry, Medical University of Bialystok, 15-089 Bialystok, Poland; anna.zalewska1@umb.edu.pl
[5] Department of Restorative Dentistry, Medical University of Bialystok, 15-089 Bialystok, Poland
[6] Department of Chemical Sciences, University of Catania, 95125 Catania, Italy; alessiadistefano92@tiscali.it
* Correspondence: bozena.antonowicz@umb.edu.pl (B.A.); m.borowska@pb.edu.pl (M.B.)

Table S1. Texture analysis approaches and their features.

Features extractors	Name of features
FOS	mean, median, minimum, maximum, 10th percentile (10Perc), 90th percentile (90Perc), variance, root mean squared (RMS), kurtosis, skewness, uniformity, range, interquartile range (IR), mean absolute deviation (MAD), robust mean absolute deviation (RMAD), energy, total energy (TE), and entropy
GLCM	autocorrelation, cluster prominence (CP), cluster shade (CS), cluster tendency (CT), contrast, correlation, difference average (DA), difference entropy (DE), difference variance (DV), inverse difference (Id), inverse difference moment (Idm), inverse difference moment normalized (Idmn), inverse difference normalized (Idn), informational measure of correlation 1 (Imc 1), informational measure of correlation 2 (Imc 2), inverse variance (IV), joint average (JA), joint energy (JE), joint entropy (JEn), maximal correlation coefficient (MCC), maximum probability (MP), sum average (SA), sum entropy (SE), and sum of squares (SS)
GLDM	dependence entropy (DEn), dependence non-uniformity (DN), dependence non-uniformity normalized (DNN), dependence variance (DV), gray level non-uniformity (GLN), gray level variance (GLV), high gray level emphasis (HGLE), large dependence emphasis (LDE), large dependence high gray level emphasis (LDHGLE), large dependence low gray level emphasis (LDLGLE), low gray level emphasis (LGLE), small dependence emphasis (SDE), small dependence high gray level emphasis (SDHGLE), and small dependence low gray level emphasis (SDLGLE)
GLRLM	gray level non-uniformity (GLN), gray level non-uniformity normalized (GLNN), gray level variance (GLV), high gray level run emphasis (HGLRE), long run emphasis (LRE), long run high gray level emphasis (LRHGLE), long run low gray level emphasis (LRLGLE), low gray level run emphasis (LGLRE), run entropy (RE), run length non-uniformity (RLN), run length non-uniformity normalized (RLNN), run percentage (RP), run variance (RV), short run emphasis (SRE), short run high gray level emphasis (SRHGLE), and short run low gray level emphasis (SRLGLE)
GLSZM	gray level non-uniformity (GLN), gray level non-uniformity normalized (GLNN), gray level variance (GLV), high gray level zone emphasis (HGLZE), large area emphasis (LAE), large area high gray level emphasis (LAHGLE), large area low gray level emphasis (LALGLE), low gray level zone emphasis (LGLZE), size-zone non-uniformity (SZN), size-zone non-uniformity normalized (SZNN), small area emphasis (SAE), small area high gray level emphasis (SAHGLE), small area low gray level emphasis (SALGLE), zone entropy (ZE), zone percentage (ZP), and zone variance (ZV)
NGTDM	busyness, coarseness, complexity, contrast, and strength

Table S2. The values (mean ± SD) of features of First Order Statistics (FOS), Gray Level Co-occurrence Matrix (GLCM), Neighbouring Gray Tone Difference Matrix (NGTDM), Gray Level Dependence Matrix (GLDM), Gray Level Run Length Matrix (GLRLM), and Gray Level Size Zone Matrix (GLSZM) of output images, filtrated by Laplacian Sharpening filter, Mean filter, and Median filter, compared between control group (Group A) and study group (Group B) of maxilla. Statistical significance was set at $p < 0.05$.

	Features	LS			Mean			Median		
		Group A	Group B	p	Group A	Group B	p	Group A	Group B	p
		mean ± SD	mean ± SD		mean ± SD	mean ± SD		mean ± SD	mean ± SD	
FOS	FOS_10Perc	126.79 ± 28.46	129.35 ± 20.41	8.54E-01	134.87 ± 27.32	136.00 ± 19.97	5.74E-01	135.23 ± 27.36	136.40 ± 20.01	5.81E-01
	FOS_90Perc	164.93 ± 27.26	178.43 ± 17.21	4.03E-03	157.81 ± 28.85	171.83 ± 18.18	1.79E-02	158.37 ± 28.77	172.28 ± 18.14	1.75E-02
	FOS_Energy	99608467.27 ± 49009373.87	97667358.23 ± 44280453.54	7.46E-01	98973735.00 ± 48961197.79	97129048.73 ± 44031135.56	7.46E-01	99569778.77 ± 49193781.59	97671850.47 ± 44254178.29	7.46E-01
	FOS_Entropy	1.44 ± 0.26	1.71 ± 0.27	2.00E-05	0.80 ± 0.38	1.25 ± 0.34	6.82E-06	0.81 ± 0.38	1.25 ± 0.34	8.63E-06
	FOS_IR	20.06 ± 5.03	26.00 ± 7.90	1.23E-04	12.52 ± 6.23	19.63 ± 10.52	1.23E-03	12.79 ± 6.33	19.93 ± 10.53	2.05E-03
	FOS_Kurtosis	3.27 ± 0.43	3.10 ± 0.47	1.47E-01	2.68 ± 0.53	2.61 ± 0.68	7.04E-01	2.68 ± 0.52	2.61 ± 0.67	6.65E-01
	FOS_Maximum	204.27 ± 24.51	223.97 ± 17.07	6.90E-05	168.53 ± 27.76	184.07 ± 16.06	2.02E-03	169.00 ± 27.62	184.67 ± 16.03	2.55E-03
	FOS_MAD	11.98 ± 2.73	15.28 ± 4.07	4.16E-05	7.15 ± 2.99	11.21 ± 4.90	2.37E-05	7.19 ± 2.98	11.23 ± 4.89	2.69E-05
	FOS_Mean	146.18 ± 27.58	154.91 ± 18.33	1.19E-01	146.18 ± 27.64	155.06 ± 18.34	1.19E-01	146.64 ± 27.64	155.50 ± 18.34	1.19E-01
	FOS_Median	146.27 ± 27.81	155.87 ± 19.11	8.12E-02	146.17 ± 27.78	156.13 ± 19.76	6.60E-02	146.53 ± 27.72	156.62 ± 19.82	6.60E-02
	FOS_Minimum	96.77 ± 30.74	97.23 ± 27.50	8.71E-01	124.17 ± 27.20	123.33 ± 20.99	4.77E-01	124.33 ± 26.98	123.63 ± 20.86	4.89E-01
	FOS_Range	107.50 ± 27.88	126.73 ± 27.46	5.38E-03	44.37 ± 13.35	60.73 ± 19.37	1.92E-05	44.67 ± 13.19	61.03 ± 19.10	1.76E-05
	FOS_RMAD	8.44 ± 2.03	10.89 ± 3.11	7.06E-05	5.38 ± 2.49	8.49 ± 4.02	2.83E-04	5.45 ± 2.53	8.54 ± 3.99	2.32E-04
	FOS_RMS	147.05 ± 27.24	156.18 ± 18.13	1.00E-01	146.49 ± 27.59	155.75 ± 18.30	1.00E-01	146.95 ± 27.59	156.19 ± 18.31	1.05E-01
	FOS_Skewness	0.00 ± 0.24	-0.07 ± 0.34	2.65E-01	0.10 ± 0.42	-0.04 ± 0.62	2.92E-01	0.10 ± 0.41	-0.04 ± 0.61	2.98E-01
	FOS_TE	99608467.27 ± 49009373.87	97667358.23 ± 44280453.54	7.46E-01	98973735.00 ± 48961197.79	97129048.73 ± 44031135.56	7.46E-01	99569778.77 ± 49193781.59	97671850.47 ± 44254178.29	7.46E-01
	FOS_Uniformity	0.44 ± 0.09	0.36 ± 0.07	7.91E-05	0.66 ± 0.18	0.49 ± 0.12	7.06E-05	0.65 ± 0.18	0.49 ± 0.12	1.37E-04
	FOS_Variance	239.46 ± 100.95	387.31 ± 200.26	7.91E-05	87.27 ± 71.35	212.91 ± 187.67	2.08E-05	87.99 ± 71.36	213.54 ± 187.63	2.08E-05
GLCM	GLCM_Autocorrelation	9.88 ± 5.83	12.21 ± 5.62	1.46E-01	3.99 ± 2.02	5.83 ± 3.19	7.11E-03	4.20 ± 2.08	5.90 ± 3.21	1.28E-02
	GLCM_CP	4.31 ± 3.28	14.31 ± 18.35	6.92E-06	1.53 ± 1.94	9.75 ± 19.12	6.92E-06	1.54 ± 1.95	9.71 ± 19.00	4.42E-06
	GLCM_CS	-0.06 ± 0.39	-0.96 ± 2.51	2.80E-01	0.05 ± 0.44	-1.14 ± 2.82	1.45E-02	0.05 ± 0.44	-1.13 ± 2.80	1.64E-02

	GLCM_CT	1.13 ± 0.48	2.00 ± 1.20	1.06E-05	0.64 ± 0.49	1.52 ± 1.28	1.06E-05	0.64 ± 0.49	1.51 ± 1.27	1.22E-05
	GLCM_Contrast	0.69 ± 0.27	0.78 ± 0.22	2.12E-01	0.14 ± 0.07	0.17 ± 0.07	2.86E-02	0.14 ± 0.07	0.18 ± 0.07	3.91E-02
	GLCM_Correlation	0.22 ± 0.17	0.38 ± 0.19	2.56E-04	0.52 ± 0.24	0.73 ± 0.16	1.11E-04	0.51 ± 0.25	0.72 ± 0.16	8.86E-05
	GLCM_DA	0.55 ± 0.14	0.60 ± 0.12	1.19E-01	0.14 ± 0.07	0.17 ± 0.07	2.87E-02	0.14 ± 0.07	0.18 ± 0.07	3.91E-02
	GLCM_DE	1.24 ± 0.19	1.31 ± 0.15	1.19E-01	0.55 ± 0.19	0.64 ± 0.15	3.09E-02	0.56 ± 0.19	0.64 ± 0.15	4.33E-02
	GLCM_DV	0.37 ± 0.11	0.40 ± 0.09	1.52E-01	0.11 ± 0.05	0.14 ± 0.04	3.09E-02	0.12 ± 0.05	0.14 ± 0.04	4.31E-02
	GLCM_Id	0.75 ± 0.05	0.73 ± 0.04	1.09E-01	0.93 ± 0.03	0.91 ± 0.03	2.87E-02	0.93 ± 0.03	0.91 ± 0.03	3.91E-02
	GLCM_Idm	0.74 ± 0.06	0.72 ± 0.05	1.29E-01	0.93 ± 0.03	0.91 ± 0.03	2.87E-02	0.93 ± 0.03	0.91 ± 0.03	3.91E-02
	GLCM_Idmn	0.98 ± 0.01	0.98 ± 0.00	1.64E-02	0.98 ± 0.01	0.98 ± 0.01	8.39E-01	0.98 ± 0.01	0.98 ± 0.01	9.35E-01
	GLCM_Idn	0.91 ± 0.02	0.92 ± 0.01	2.07E-01	0.96 ± 0.02	0.96 ± 0.02	3.28E-01	0.96 ± 0.02	0.96 ± 0.02	2.62E-01
	GLCM_Imc1	-0.05 ± 0.06	-0.10 ± 0.08	1.13E-03	-0.27 ± 0.17	-0.44 ± 0.16	4.18E-04	-0.27 ± 0.18	-0.44 ± 0.15	3.13E-04
	GLCM_Imc2	0.28 ± 0.19	0.47 ± 0.19	6.29E-05	0.53 ± 0.26	0.77 ± 0.16	2.08E-05	0.53 ± 0.26	0.77 ± 0.15	2.69E-05
	GLCM_IV	0.44 ± 0.06	0.46 ± 0.04	8.41E-02	0.14 ± 0.07	0.17 ± 0.07	2.87E-02	0.14 ± 0.07	0.18 ± 0.07	3.91E-02
	GLCM_JA	3.00 ± 0.90	3.36 ± 0.81	9.77E-02	1.90 ± 0.52	2.27 ± 0.61	5.73E-03	1.95 ± 0.53	2.28 ± 0.60	1.73E-02
	GLCM_JE	0.21 ± 0.09	0.16 ± 0.06	5.05E-04	0.56 ± 0.21	0.39 ± 0.13	2.09E-04	0.55 ± 0.21	0.38 ± 0.13	3.13E-04
	GLCM_JEn	2.78 ± 0.50	3.22 ± 0.46	3.13E-04	1.30 ± 0.59	1.89 ± 0.45	3.00E-05	1.32 ± 0.58	1.90 ± 0.46	4.30E-05
	GLCM_MCC	0.24 ± 0.17	0.42 ± 0.20	7.06E-05	0.53 ± 0.25	0.77 ± 0.16	1.60E-05	0.53 ± 0.26	0.77 ± 0.16	1.82E-05
	GLCM_MP	0.36 ± 0.12	0.28 ± 0.08	9.52E-04	0.69 ± 0.18	0.54 ± 0.14	5.48E-04	0.68 ± 0.18	0.54 ± 0.14	8.53E-04
	GLCM_SA	5.99 ± 1.81	6.72 ± 1.62	9.77E-02	3.80 ± 1.03	4.53 ± 1.21	5.73E-03	3.90 ± 1.05	4.57 ± 1.21	1.73E-02
	GLCM_SE	2.04 ± 0.31	2.39 ± 0.33	4.65E-06	1.17 ± 0.53	1.72 ± 0.42	2.04E-05	1.18 ± 0.53	1.72 ± 0.43	2.80E-05
	GLCM_SS	0.46 ± 0.16	0.69 ± 0.31	3.90E-05	0.19 ± 0.13	0.42 ± 0.32	1.22E-05	0.20 ± 0.13	0.42 ± 0.32	1.22E-05
**GLDM**	GLDM_DEn	6.01 ± 0.33	6.28 ± 0.37	1.58E-03	4.77 ± 0.96	5.80 ± 0.42	4.40E-06	4.82 ± 0.96	5.81 ± 0.43	9.31E-06
	GLDM_DNU	144.51 ± 38.55	137.73 ± 50.68	4.71E-01	676.87 ± 529.50	274.98 ± 213.04	1.23E-04	650.92 ± 521.11	270.37 ± 210.07	3.45E-04
	GLDM_DNUN	0.03 ± 0.00	0.04 ± 0.01	3.45E-02	0.15 ± 0.10	0.06 ± 0.03	1.06E-05	0.14 ± 0.10	0.06 ± 0.03	1.82E-05
	GLDM_DV	79.83 ± 22.65	62.50 ± 17.51	3.12E-03	93.07 ± 16.55	85.86 ± 17.20	1.15E-01	93.90 ± 17.07	86.30 ± 17.44	9.86E-02
	GLDM_GLNU	1903.59 ± 654.64	1394.70 ± 562.36	1.04E-03	2840.71 ± 1089.30	1885.32 ± 811.67	1.37E-04	2818.58 ± 1067.29	1887.40 ± 812.39	2.09E-04
	GLDM_GLV	0.46 ± 0.16	0.70 ± 0.32	2.69E-05	0.20 ± 0.13	0.43 ± 0.32	5.14E-06	0.20 ± 0.13	0.43 ± 0.32	5.97E-06
	GLDM_HGLE	10.27 ± 5.91	12.56 ± 5.63	1.64E-01	4.08 ± 2.02	5.90 ± 3.16	8.14E-03	4.29 ± 2.08	5.97 ± 3.17	1.45E-02
	GLDM_LDE	459.75 ± 152.94	331.24 ± 100.56	6.40E-04	1092.29 ± 148.15	839.11 ± 199.57	5.97E-06	1083.31 ± 151.20	834.93 ± 199.19	9.22E-06

	GLDM_LDHGLE	4289.90 ± 2198.24	3963.03 ± 1711.16	5.70E-01	4384.98 ± 2414.95	5215.74 ± 3631.64	4.90E-01	4547.17 ± 2416.87	5239.95 ± 3615.08	6.55E-01
	GLDM_LDLGLE	79.67 ± 60.75	45.50 ± 37.09	2.62E-02	467.49 ± 341.72	265.69 ± 182.08	1.37E-02	442.28 ± 338.16	257.57 ± 177.72	1.97E-02
	GLDM_LGLE	0.19 ± 0.13	0.15 ± 0.09	1.84E-01	0.43 ± 0.27	0.34 ± 0.17	2.53E-01	0.41 ± 0.27	0.33 ± 0.17	3.28E-01
	GLDM_SDE	0.03 ± 0.01	0.04 ± 0.01	1.23E-03	0.01 ± 0.00	0.01 ± 0.00	2.25E-01	0.01 ± 0.00	0.01 ± 0.00	1.02E-01
	GLDM_SDHGLE	0.38 ± 0.27	0.57 ± 0.30	1.45E-02	0.03 ± 0.02	0.04 ± 0.03	3.84E-02	0.04 ± 0.02	0.05 ± 0.03	5.77E-02
	GLDM_SDLGLE	0.01 ± 0.00	0.01 ± 0.00	2.89E-01	0.00 ± 0.00	0.00 ± 0.00	4.28E-01	0.00 ± 0.00	0.00 ± 0.00	2.53E-01
GLRLM	GLRLM_GLNU	652.00 ± 189.29	563.38 ± 229.35	4.28E-02	137.69 ± 42.69	132.16 ± 41.04	4.77E-01	142.30 ± 47.60	133.78 ± 41.70	3.39E-01
	GLRLM_GLNUN	0.35 ± 0.04	0.31 ± 0.05	8.79E-04	0.49 ± 0.11	0.41 ± 0.09	3.45E-05	0.49 ± 0.11	0.41 ± 0.09	2.69E-05
	GLRLM_GLV	0.66 ± 0.17	0.88 ± 0.31	8.22E-05	0.33 ± 0.14	0.55 ± 0.34	9.22E-06	0.33 ± 0.14	0.55 ± 0.34	3.79E-06
	GLRLM_HGLRE	10.42 ± 5.87	12.57 ± 5.64	1.91E-01	4.34 ± 2.05	5.73 ± 2.93	2.77E-02	4.55 ± 2.10	5.83 ± 3.01	5.49E-02
	GLRLM_LRE	12.75 ± 8.62	9.01 ± 4.35	3.64E-02	657.30 ± 516.63	289.21 ± 140.22	4.41E-05	629.10 ± 503.23	282.87 ± 140.02	1.11E-04
	GLRLM_LRHGLE	112.16 ± 67.42	101.52 ± 38.17	5.16E-01	2418.45 ± 1777.11	1855.38 ± 1677.82	1.52E-01	2406.18 ± 1781.82	1815.93 ± 1610.39	1.14E-01
	GLRLM_LRLGLE	2.32 ± 2.28	1.34 ± 1.22	6.67E-02	310.51 ± 494.88	92.55 ± 89.55	1.13E-03	284.66 ± 464.70	88.64 ± 87.02	1.46E-03
	GLRLM_LGLRE	0.22 ± 0.15	0.16 ± 0.10	1.40E-01	0.43 ± 0.21	0.37 ± 0.15	2.21E-01	0.41 ± 0.21	0.36 ± 0.15	3.28E-01
	GLRLM_RE	3.75 ± 0.23	3.86 ± 0.23	6.32E-02	5.51 ± 0.33	5.62 ± 0.48	1.83E-01	5.49 ± 0.34	5.62 ± 0.48	1.50E-01
	GLRLM_RLNU	681.71 ± 275.97	692.95 ± 351.73	9.52E-01	16.89 ± 8.38	18.62 ± 6.71	2.62E-01	18.17 ± 9.94	19.33 ± 7.43	3.71E-01
	GLRLM_RLNUN	0.34 ± 0.06	0.36 ± 0.05	2.35E-01	0.05 ± 0.01	0.06 ± 0.01	7.06E-01	0.06 ± 0.01	0.06 ± 0.01	8.55E-01
	GLRLM_RP	0.44 ± 0.09	0.48 ± 0.07	8.41E-02	0.07 ± 0.02	0.09 ± 0.03	1.72E-03	0.07 ± 0.02	0.09 ± 0.03	2.02E-03
	GLRLM_RV	6.83 ± 5.96	4.23 ± 2.65	2.48E-02	283.82 ± 156.22	120.84 ± 62.52	2.35E-06	274.90 ± 156.64	119.73 ± 62.55	5.14E-06
	GLRLM_SRE	0.60 ± 0.06	0.61 ± 0.05	2.62E-01	0.14 ± 0.04	0.13 ± 0.03	6.06E-02	0.14 ± 0.04	0.13 ± 0.03	1.64E-01
	GLRLM_SRHGLE	6.49 ± 4.05	7.96 ± 3.88	1.84E-01	0.70 ± 0.44	0.71 ± 0.41	9.84E-01	0.72 ± 0.44	0.75 ± 0.52	9.52E-01
	GLRLM_SRLGLE	0.14 ± 0.09	0.11 ± 0.06	1.58E-01	0.06 ± 0.03	0.05 ± 0.03	2.64E-01	0.06 ± 0.03	0.05 ± 0.03	4.66E-01
GLSZM	GLSZM_GLNU	115.78 ± 46.29	89.51 ± 40.19	8.62E-03	6.88 ± 4.97	5.53 ± 2.61	3.39E-01	6.71 ± 4.42	5.40 ± 2.44	3.28E-01
	GLSZM_GLNUN	0.31 ± 0.08	0.25 ± 0.05	2.56E-04	0.52 ± 0.14	0.40 ± 0.11	7.91E-05	0.50 ± 0.13	0.40 ± 0.10	1.80E-03
	GLSZM_GLV	1.36 ± 0.34	1.64 ± 0.40	3.59E-03	0.49 ± 0.29	0.71 ± 0.46	3.10E-02	0.50 ± 0.29	0.74 ± 0.45	2.48E-02
	GLSZM_HGLZE	11.14 ± 5.77	13.50 ± 5.73	1.64E-01	4.82 ± 2.68	5.56 ± 3.32	4.65E-01	4.98 ± 2.47	5.84 ± 3.70	6.26E-01
	GLSZM_LAE	19074.69 ± 12132.94	8104.37 ± 5569.95	2.69E-05	1365165.94 ± 1362525.50	442802.59 ± 292768.05	1.06E-05	1323191.77 ± 1353625.22	448053.99 ± 293747.21	2.37E-05
	GLSZM_LAHGLE	153134.19 ± 91098.80	91694.42 ± 59863.52	2.73E-03	4892937.87 ± 4410244.56	3057001.39 ± 3416750.85	2.93E-02	4895682.32 ± 4530700.26	3117998.84 ± 3539777.92	1.00E-01

	GLSZM_LALGLE	3481.96 ± 3711.03	1144.82 ± 1408.10	4.34E-03	664963.30 ± 1238085.85	129266.21 ± 149974.41	2.32E-04	612632.06 ± 1158828.05	127592.77 ± 143391.53	5.55E-04
	GLSZM_LGLZE	0.28 ± 0.18	0.21 ± 0.12	1.19E-01	0.46 ± 0.20	0.44 ± 0.19	6.93E-01	0.43 ± 0.18	0.44 ± 0.19	9.27E-01
	GLSZM_SZNU	110.27 ± 44.94	116.43 ± 60.45	8.55E-01	1.53 ± 0.49	1.43 ± 0.58	1.63E-01	1.55 ± 0.59	1.27 ± 0.37	2.63E-02
	GLSZM_SZNUN	0.29 ± 0.03	0.30 ± 0.03	3.71E-02	0.16 ± 0.11	0.11 ± 0.04	1.55E-02	0.15 ± 0.09	0.10 ± 0.03	2.56E-03
	GLSZM_SAE	0.55 ± 0.03	0.57 ± 0.03	1.61E-02	0.18 ± 0.10	0.15 ± 0.10	1.14E-01	0.13 ± 0.10	0.11 ± 0.07	4.77E-01
	GLSZM_SAHGLE	6.42 ± 3.24	8.01 ± 3.48	1.00E-01	0.93 ± 0.94	0.97 ± 1.01	7.15E-01	0.65 ± 0.58	0.66 ± 0.73	9.19E-01
	GLSZM_SALGLE	0.15 ± 0.08	0.13 ± 0.07	2.71E-01	0.08 ± 0.08	0.06 ± 0.05	2.45E-01	0.06 ± 0.05	0.04 ± 0.04	2.80E-01
	GLSZM_ZE	4.20 ± 0.35	4.48 ± 0.26	7.98E-04	3.22 ± 0.89	3.52 ± 0.52	6.45E-02	3.27 ± 0.83	3.57 ± 0.52	5.82E-02
	GLSZM_ZP	0.09 ± 0.03	0.10 ± 0.03	2.22E-01	0.00 ± 0.00	0.00 ± 0.00	2.77E-02	0.00 ± 0.00	0.00 ± 0.00	8.03E-02
	GLSZM_ZV	18894.21 ± 12054.83	7966.25 ± 5511.05	2.08E-05	975615.65 ± 821871.24	333370.57 ± 239315.07	1.68E-06	1007020.81 ± 885541.24	339607.84 ± 242426.07	5.97E-06
**NGTDM**	NGTDM_Busyness	44.91 ± 33.01	26.92 ± 18.64	1.64E-02	105.08 ± 167.17	99.35 ± 162.36	8.08E-01	112.74 ± 214.86	78.18 ± 121.37	4.16E-01
	NGTDM_Coarseness	0.00 ± 0.00	0.00 ± 0.00	3.45E-02	0.01 ± 0.01	0.01 ± 0.00	8.24E-01	0.01 ± 0.01	0.01 ± 0.00	7.77E-01
	NGTDM_Complexity	5.97 ± 3.89	8.54 ± 3.87	1.45E-02	0.47 ± 0.34	0.77 ± 0.55	6.19E-03	0.51 ± 0.36	0.79 ± 0.54	1.55E-02
	NGTDM_Contrast	0.02 ± 0.01	0.02 ± 0.01	3.22E-01	0.01 ± 0.01	0.02 ± 0.02	3.64E-02	0.01 ± 0.01	0.02 ± 0.02	2.93E-02
	NGTDM_Strength	0.02 ± 0.01	0.03 ± 0.02	1.70E-04	0.01 ± 0.01	0.02 ± 0.01	4.27E-02	0.01 ± 0.01	0.02 ± 0.01	2.77E-02

Table S3. The values (mean ± SD) of features of First Order Statistics (FOS), Gray Level Co-occurrence Matrix (GLCM), Neighbouring Gray Tone Difference Matrix (NGTDM), Gray Level Dependence Matrix (GLDM), Gray Level Run Length Matrix (GLRLM), and Gray Level Size Zone Matrix (GLSZM) of output images, filtrated by Laplacian Sharpening filter, Mean filter, and Median filter, compared between control group (Group C) and study group (Group D) of mandible. Statistical significance was set at $p < 0.05$.

	Features	LS			Mean			Median		
		Group C mean ± SD	Group D mean ± SD	p	Group C mean ± SD	Group D mean ± SD	p	Group C mean ± SD	Group D mean ± SD	p
FOS	FOS_10Perc	123.69 ± 31.01	128.13 ± 23.40	5.63E-01	131.12 ± 30.27	137.19 ± 21.34	4.16E-01	131.50 ± 30.37	137.65 ± 21.34	4.13E-01
	FOS_90Perc	158.73 ± 22.17	168.80 ± 16.15	3.45E-02	151.31 ± 24.02	161.50 ± 18.44	4.97E-02	151.85 ± 24.03	161.88 ± 18.48	5.27E-02
	FOS_Energy	221689512.77 ± 127415620.91	145984175.35 ± 83945140.28	1.66E-04	220316306.35 ± 127765283.55	145077484.69 ± 84036115.98	1.66E-04	221674709.96 ± 128361180.21	145924440.54 ± 84445698.71	1.66E-04
	FOS_Entropy	1.33 ± 0.40	1.51 ± 0.32	1.18E-01	0.68 ± 0.50	0.87 ± 0.36	1.23E-01	0.69 ± 0.50	0.87 ± 0.36	1.16E-01
	FOS_IR	18.42 ± 6.84	21.14 ± 6.41	1.66E-01	11.31 ± 7.47	13.15 ± 7.39	2.00E-01	11.31 ± 7.29	13.23 ± 7.61	2.27E-01
	FOS_Kurtosis	3.38 ± 0.53	3.53 ± 0.65	2.61E-01	2.52 ± 0.48	2.78 ± 0.78	1.29E-01	2.52 ± 0.47	2.78 ± 0.78	1.43E-01
	FOS_Maximum	193.88 ± 17.00	217.46 ± 17.55	7.29E-07	160.69 ± 21.98	172.81 ± 18.34	1.24E-02	161.69 ± 21.77	173.31 ± 18.24	1.57E-02
	FOS_MAD	11.05 ± 3.75	12.80 ± 3.44	1.18E-01	6.28 ± 3.77	7.52 ± 3.39	1.43E-01	6.32 ± 3.76	7.54 ± 3.39	1.43E-01
	FOS_Mean	141.23 ± 26.04	148.68 ± 19.49	2.22E-01	141.27 ± 26.07	148.78 ± 19.54	2.18E-01	141.71 ± 26.06	149.23 ± 19.53	2.19E-01
	FOS_Median	141.23 ± 25.85	148.65 ± 19.61	2.27E-01	141.27 ± 25.35	147.69 ± 20.15	2.97E-01	141.77 ± 25.45	148.21 ± 20.06	2.98E-01
	FOS_Minimum	85.35 ± 39.20	84.62 ± 28.97	9.39E-01	121.58 ± 32.99	126.00 ± 21.76	5.82E-01	121.73 ± 32.97	126.23 ± 21.81	5.76E-01
	FOS_Range	108.54 ± 34.43	132.85 ± 35.69	2.66E-02	39.12 ± 17.31	46.81 ± 14.76	1.27E-01	39.96 ± 17.48	47.08 ± 14.81	1.58E-01
	FOS_RMAD	7.82 ± 2.81	8.98 ± 2.55	1.51E-01	4.91 ± 3.08	5.72 ± 3.00	2.08E-01	4.93 ± 3.14	5.72 ± 2.94	2.08E-01
	FOS_RMS	142.08 ± 25.47	149.69 ± 18.91	1.99E-01	141.57 ± 25.86	149.13 ± 19.43	2.10E-01	142.01 ± 25.86	149.57 ± 19.42	2.10E-01
	FOS_Skewness	-0.04 ± 0.13	-0.06 ± 0.25	7.84E-01	0.02 ± 0.33	0.07 ± 0.55	6.55E-01	0.02 ± 0.33	0.07 ± 0.54	6.70E-01
	FOS_TE	221689512.77 ± 127415620.91	145984175.35 ± 83945140.28	1.66E-04	220316306.35 ± 127765283.55	145077484.69 ± 84036115.98	1.66E-04	221674709.96 ± 128361180.21	145924440.54 ± 84445698.71	1.66E-04
	FOS_Uniformity	0.48 ± 0.14	0.43 ± 0.10	6.30E-02	0.71 ± 0.22	0.63 ± 0.16	9.93E-02	0.71 ± 0.22	0.63 ± 0.16	1.05E-01
	FOS_Variance	215.20 ± 136.94	281.24 ± 140.98	5.94E-02	74.44 ± 83.80	97.76 ± 77.03	1.65E-01	75.01 ± 83.91	98.22 ± 77.17	1.65E-01
GLCM	GLCM_Autocorrelation	11.01 ± 4.86	13.54 ± 6.71	1.29E-01	3.71 ± 2.10	4.38 ± 1.93	1.29E-01	3.77 ± 2.12	4.46 ± 1.96	2.37E-01
	GLCM_CP	4.13 ± 4.93	6.10 ± 4.93	5.94E-02	1.57 ± 2.05	2.06 ± 2.36	3.40E-01	1.58 ± 2.06	2.13 ± 2.40	2.69E-01
	GLCM_CS	-0.07 ± 0.19	-0.02 ± 0.65	1.57E-01	-0.07 ± 0.34	0.19 ± 0.80	2.92E-02	-0.06 ± 0.33	0.22 ± 0.80	3.57E-02

	GLCM_CT	1.04 ± 0.66	1.31 ± 0.60	4.35E-02	0.59 ± 0.56	0.73 ± 0.42	2.08E-01	0.59 ± 0.56	0.75 ± 0.43	1.73E-01
	GLCM_Contrast	0.63 ± 0.29	0.77 ± 0.39	1.44E-01	0.09 ± 0.07	0.11 ± 0.06	2.68E-01	0.09 ± 0.07	0.11 ± 0.06	3.68E-01
	GLCM_Correlation	0.20 ± 0.17	0.26 ± 0.20	3.03E-01	0.50 ± 0.32	0.65 ± 0.25	4.07E-02	0.50 ± 0.32	0.65 ± 0.24	3.12E-02
	GLCM_DA	0.51 ± 0.18	0.58 ± 0.20	2.29E-01	0.09 ± 0.07	0.11 ± 0.06	2.68E-01	0.09 ± 0.07	0.11 ± 0.06	3.68E-01
	GLCM_DE	1.17 ± 0.26	1.27 ± 0.27	2.00E-01	0.39 ± 0.23	0.46 ± 0.20	1.81E-01	0.41 ± 0.23	0.47 ± 0.20	3.05E-01
	GLCM_DV	0.34 ± 0.12	0.40 ± 0.15	1.17E-01	0.08 ± 0.05	0.09 ± 0.05	2.47E-01	0.08 ± 0.05	0.10 ± 0.05	3.31E-01
	GLCM_Id	0.76 ± 0.07	0.74 ± 0.08	2.37E-01	0.96 ± 0.03	0.95 ± 0.03	2.68E-01	0.95 ± 0.04	0.94 ± 0.03	3.68E-01
	GLCM_Idm	0.76 ± 0.08	0.73 ± 0.08	2.79E-01	0.96 ± 0.03	0.95 ± 0.03	2.68E-01	0.95 ± 0.04	0.94 ± 0.03	3.68E-01
	GLCM_Idmn	0.98 ± 0.01	0.98 ± 0.01	1.29E-01	0.99 ± 0.01	0.99 ± 0.01	6.35E-01	0.99 ± 0.01	0.99 ± 0.01	7.08E-01
	GLCM_Idn	0.92 ± 0.02	0.92 ± 0.02	8.42E-01	0.98 ± 0.02	0.97 ± 0.02	4.37E-01	0.97 ± 0.02	0.97 ± 0.02	4.37E-01
	GLCM_Imc1	-0.04 ± 0.08	-0.07 ± 0.09	3.40E-01	-0.31 ± 0.23	-0.41 ± 0.21	6.30E-02	-0.30 ± 0.22	-0.41 ± 0.20	4.07E-02
	GLCM_Imc2	0.25 ± 0.20	0.34 ± 0.20	1.11E-01	0.50 ± 0.34	0.65 ± 0.25	7.51E-02	0.49 ± 0.34	0.65 ± 0.26	5.59E-02
	GLCM_IV	0.41 ± 0.11	0.43 ± 0.09	3.94E-01	0.09 ± 0.07	0.11 ± 0.06	2.68E-01	0.09 ± 0.07	0.11 ± 0.06	3.68E-01
	GLCM_JA	3.21 ± 0.79	3.55 ± 0.93	1.76E-01	1.82 ± 0.54	2.00 ± 0.49	2.15E-01	1.83 ± 0.54	2.02 ± 0.49	2.14E-01
	GLCM_JE	0.27 ± 0.17	0.21 ± 0.12	1.99E-01	0.65 ± 0.26	0.56 ± 0.19	9.41E-02	0.64 ± 0.26	0.55 ± 0.19	1.11E-01
	GLCM_JEn	2.58 ± 0.77	2.89 ± 0.67	1.64E-01	1.04 ± 0.73	1.28 ± 0.53	1.16E-01	1.06 ± 0.73	1.30 ± 0.53	1.36E-01
	GLCM_MCC	0.21 ± 0.19	0.29 ± 0.20	1.50E-01	0.52 ± 0.33	0.68 ± 0.26	3.82E-02	0.51 ± 0.33	0.68 ± 0.26	2.92E-02
	GLCM_MP	0.40 ± 0.18	0.35 ± 0.15	1.36E-01	0.74 ± 0.22	0.68 ± 0.17	1.43E-01	0.74 ± 0.22	0.68 ± 0.17	1.43E-01
	GLCM_SA	6.42 ± 1.59	7.09 ± 1.85	1.76E-01	3.64 ± 1.08	4.00 ± 0.98	2.15E-01	3.67 ± 1.08	4.03 ± 0.98	2.14E-01
	GLCM_SE	1.90 ± 0.51	2.10 ± 0.38	1.55E-01	0.95 ± 0.67	1.17 ± 0.48	1.11E-01	0.97 ± 0.67	1.19 ± 0.48	1.23E-01
	GLCM_SS	0.42 ± 0.22	0.52 ± 0.22	1.41E-01	0.17 ± 0.15	0.21 ± 0.11	1.81E-01	0.17 ± 0.15	0.22 ± 0.11	1.57E-01
**GLDM**	GLDM_DEn	5.80 ± 0.60	5.94 ± 0.46	5.15E-01	4.01 ± 1.21	4.90 ± 0.78	7.26E-03	4.09 ± 1.22	4.93 ± 0.77	1.09E-02
	GLDM_DNU	427.91 ± 321.75	225.20 ± 93.05	1.26E-04	3016.50 ± 2143.03	997.85 ± 981.00	3.19E-05	2830.43 ± 2059.11	975.07 ± 957.09	4.41E-05
	GLDM_DNUN	0.04 ± 0.02	0.04 ± 0.01	3.40E-01	0.27 ± 0.15	0.13 ± 0.09	1.17E-03	0.25 ± 0.15	0.13 ± 0.08	2.66E-03
	GLDM_DV	80.34 ± 21.69	71.04 ± 30.75	1.87E-01	72.76 ± 20.66	81.12 ± 22.14	1.73E-01	74.92 ± 21.13	80.99 ± 21.60	3.67E-01
	GLDM_GLNU	5126.18 ± 2724.04	2704.21 ± 1490.13	1.60E-05	7356.45 ± 3401.33	3933.65 ± 1867.18	2.09E-05	7325.67 ± 3367.16	3904.01 ± 1860.65	1.59E-05
	GLDM_GLV	0.42 ± 0.23	0.53 ± 0.23	1.30E-01	0.17 ± 0.16	0.22 ± 0.11	1.50E-01	0.17 ± 0.16	0.22 ± 0.12	1.43E-01
	GLDM_HGLE	11.34 ± 4.98	13.89 ± 6.81	1.33E-01	3.76 ± 2.12	4.43 ± 1.92	2.51E-01	3.82 ± 2.14	4.50 ± 1.94	2.48E-01
	GLDM_LDE	546.42 ± 259.58	409.42 ± 239.04	4.07E-02	1257.85 ± 172.16	998.47 ± 288.13	5.16E-05	1246.78 ± 174.59	993.84 ± 286.36	6.03E-05

	GLDM_LDHGLE	5312.31 ± 1933.22	4872.23 ± 2372.74	3.24E-01	4646.81 ± 2543.40	4297.25 ± 2126.15	5.48E-01	4674.37 ± 2547.88	4339.68 ± 2138.64	5.68E-01
	GLDM_LDLGLE	93.98 ± 133.65	52.92 ± 59.04	2.58E-01	592.53 ± 435.10	380.27 ± 315.23	3.57E-02	575.88 ± 423.09	372.88 ± 309.15	4.07E-02
	GLDM_LGLE	0.15 ± 0.13	0.13 ± 0.09	6.89E-01	0.47 ± 0.30	0.38 ± 0.25	2.91E-01	0.46 ± 0.30	0.38 ± 0.24	2.80E-01
	GLDM_SDE	0.03 ± 0.01	0.04 ± 0.02	2.18E-03	0.01 ± 0.00	0.01 ± 0.01	1.29E-01	0.01 ± 0.00	0.01 ± 0.01	3.15E-01
	GLDM_SDHGLE	0.37 ± 0.24	0.66 ± 0.48	1.40E-02	0.02 ± 0.02	0.03 ± 0.02	1.23E-01	0.03 ± 0.02	0.03 ± 0.02	4.37E-01
	GLDM_SDLGLE	0.01 ± 0.00	0.01 ± 0.00	2.80E-01	0.00 ± 0.00	0.00 ± 0.01	4.68E-01	0.00 ± 0.00	0.00 ± 0.01	5.32E-01
**GLRLM**	GLRLM_GLNU	1527.59 ± 623.74	897.42 ± 366.32	1.06E-04	249.58 ± 103.22	192.78 ± 49.51	1.51E-02	267.28 ± 121.51	196.88 ± 49.68	1.07E-02
	GLRLM_GLNUN	0.37 ± 0.07	0.34 ± 0.05	9.13E-02	0.55 ± 0.18	0.50 ± 0.15	4.83E-01	0.54 ± 0.17	0.50 ± 0.15	6.89E-01
	GLRLM_GLV	0.61 ± 0.24	0.73 ± 0.24	1.09E-01	0.30 ± 0.18	0.35 ± 0.19	3.21E-01	0.30 ± 0.18	0.35 ± 0.19	4.03E-01
	GLRLM_HGLRE	11.41 ± 5.06	14.07 ± 6.71	1.19E-01	3.79 ± 2.09	4.72 ± 2.08	3.34E-02	3.90 ± 2.12	4.79 ± 2.11	3.82E-02
	GLRLM_LRE	33.78 ± 80.43	19.21 ± 30.63	2.37E-01	1721.65 ± 1627.65	638.66 ± 457.79	2.41E-03	1622.16 ± 1656.75	610.55 ± 433.64	6.13E-03
	GLRLM_LRHGLE	209.42 ± 315.28	175.58 ± 190.49	4.23E-01	5553.68 ± 5760.85	2509.85 ± 1964.57	3.55E-03	5480.63 ± 6173.60	2447.44 ± 1899.47	1.30E-02
	GLRLM_LRLGLE	7.74 ± 21.11	3.05 ± 6.23	3.53E-01	869.41 ± 1201.29	258.65 ± 301.38	2.04E-02	757.34 ± 1048.75	240.50 ± 271.89	2.36E-02
	GLRLM_LGLRE	0.17 ± 0.15	0.14 ± 0.10	5.32E-01	0.50 ± 0.23	0.40 ± 0.22	4.63E-02	0.48 ± 0.23	0.39 ± 0.21	5.94E-02
	GLRLM_RE	3.72 ± 0.32	3.76 ± 0.37	1.00E+00	5.79 ± 0.46	5.54 ± 0.57	4.35E-02	5.75 ± 0.44	5.53 ± 0.56	1.50E-01
	GLRLM_RLNU	1612.26 ± 1025.80	1051.82 ± 569.52	3.15E-02	29.93 ± 21.41	23.50 ± 9.45	3.67E-01	35.88 ± 27.31	24.51 ± 9.58	1.11E-01
	GLRLM_RLNUN	0.34 ± 0.07	0.37 ± 0.08	2.47E-01	0.05 ± 0.02	0.06 ± 0.02	6.53E-01	0.06 ± 0.02	0.06 ± 0.02	8.03E-01
	GLRLM_RP	0.43 ± 0.13	0.46 ± 0.13	2.37E-01	0.05 ± 0.03	0.08 ± 0.04	3.90E-03	0.05 ± 0.03	0.08 ± 0.04	1.10E-02
	GLRLM_RV	23.26 ± 62.31	11.93 ± 22.52	2.27E-01	689.83 ± 490.00	292.93 ± 196.15	3.21E-04	661.40 ± 486.84	286.86 ± 192.47	5.25E-04
	GLRLM_SRE	0.59 ± 0.07	0.62 ± 0.08	2.43E-01	0.15 ± 0.05	0.14 ± 0.04	1.57E-01	0.16 ± 0.06	0.14 ± 0.04	1.04E-01
	GLRLM_SRHGLE	7.12 ± 3.68	9.06 ± 4.72	1.07E-01	0.62 ± 0.42	0.68 ± 0.37	3.15E-01	0.68 ± 0.46	0.70 ± 0.39	5.48E-01
	GLRLM_SRLGLE	0.10 ± 0.07	0.09 ± 0.06	4.83E-01	0.08 ± 0.04	0.06 ± 0.03	1.30E-02	0.08 ± 0.04	0.06 ± 0.03	2.41E-03
**GLSZM**	GLSZM_GLNU	283.72 ± 116.80	168.04 ± 73.25	3.30E-05	14.48 ± 10.41	6.78 ± 3.38	1.17E-03	17.09 ± 15.08	7.75 ± 4.03	8.63E-03
	GLSZM_GLNUN	0.36 ± 0.14	0.30 ± 0.10	8.42E-02	0.54 ± 0.18	0.45 ± 0.11	3.13E-02	0.55 ± 0.19	0.46 ± 0.09	2.38E-02
	GLSZM_GLV	1.31 ± 0.47	1.47 ± 0.47	2.76E-01	0.40 ± 0.36	0.56 ± 0.38	7.96E-02	0.39 ± 0.34	0.54 ± 0.35	1.11E-01
	GLSZM_HGLZE	12.02 ± 5.16	14.69 ± 6.65	1.23E-01	4.12 ± 2.57	4.82 ± 2.39	1.99E-01	4.15 ± 2.55	4.86 ± 2.44	2.08E-01
	GLSZM_LAE	77131.92 ± 114060.28	32017.98 ± 49324.41	1.40E-02	5697215.75 ± 8054041.01	2469610.30 ± 2765574.10	2.54E-02	5612614.94 ± 8779051.07	2294529.39 ± 2661055.22	3.12E-02

	GLSZM_LAHGLE	568330.82 ± 509750.39	278619.75 ± 274472.07	1.04E-03	16158660.47 ± 17099732.95	8802770.08 ± 9925331.81	2.36E-02	18885940.66 ± 31708944.02	8316617.05 ± 9677587.28	3.34E-02
	GLSZM_LALGLE	17983.78 ± 40215.13	5223.37 ± 11337.52	5.25E-02	3404413.31 ± 7746899.54	1087791.69 ± 1860572.42	6.30E-02	2601325.27 ± 5104287.76	979975.61 ± 1696259.95	4.63E-02
	GLSZM_LGLZE	0.22 ± 0.18	0.17 ± 0.12	5.15E-01	0.51 ± 0.23	0.47 ± 0.19	4.73E-01	0.50 ± 0.24	0.46 ± 0.20	6.53E-01
	GLSZM_SZNU	273.68 ± 170.26	189.63 ± 106.48	5.94E-02	3.52 ± 1.99	1.75 ± 0.63	2.82E-04	3.40 ± 2.12	1.77 ± 0.53	6.13E-03
	GLSZM_SZNUN	0.30 ± 0.04	0.30 ± 0.02	6.04E-01	0.16 ± 0.10	0.14 ± 0.09	1.16E-01	0.14 ± 0.08	0.13 ± 0.09	7.84E-01
	GLSZM_SAE	0.56 ± 0.04	0.57 ± 0.03	4.68E-01	0.30 ± 0.11	0.23 ± 0.10	8.69E-03	0.22 ± 0.08	0.18 ± 0.08	2.27E-01
	GLSZM_SAHGLE	6.92 ± 3.06	8.57 ± 4.02	1.18E-01	1.22 ± 0.77	1.09 ± 0.55	8.03E-01	0.93 ± 0.83	0.92 ± 0.58	2.58E-01
	GLSZM_SALGLE	0.13 ± 0.09	0.10 ± 0.06	7.84E-01	0.14 ± 0.12	0.10 ± 0.09	9.41E-02	0.11 ± 0.08	0.08 ± 0.07	1.50E-01
	GLSZM_ZE	4.06 ± 0.51	4.30 ± 0.38	7.96E-02	3.55 ± 1.08	3.49 ± 0.80	8.81E-01	3.78 ± 1.03	3.61 ± 0.84	6.53E-01
	GLSZM_ZP	0.08 ± 0.04	0.11 ± 0.05	6.76E-02	0.00 ± 0.00	0.00 ± 0.00	7.08E-01	0.00 ± 0.00	0.00 ± 0.00	7.64E-01
	GLSZM_ZV	76831.61 ± 113673.40	31771.58 ± 48958.89	1.40E-02	4512021.26 ± 5071314.05	1981604.53 ± 2062933.13	1.63E-02	4524220.20 ± 6014770.34	1856024.16 ± 2016135.57	1.30E-02
**NGTDM**	NGTDM_Busyness	97.83 ± 126.54	40.09 ± 36.99	5.25E-04	304.20 ± 890.91	145.51 ± 385.41	1.16E-01	3053.70 ± 14735.91	198.87 ± 653.75	8.90E-02
	NGTDM_Coarseness	0.00 ± 0.00	0.00 ± 0.00	1.91E-05	0.03 ± 0.10	0.01 ± 0.02	9.80E-01	0.02 ± 0.04	0.01 ± 0.01	9.80E-01
	NGTDM_Complexity	6.11 ± 4.23	9.41 ± 6.28	3.69E-02	0.29 ± 0.39	0.40 ± 0.34	6.30E-02	0.31 ± 0.41	0.41 ± 0.35	4.63E-02
	NGTDM_Contrast	0.02 ± 0.01	0.02 ± 0.01	9.80E-01	0.01 ± 0.01	0.01 ± 0.01	4.68E-01	0.01 ± 0.01	0.01 ± 0.01	5.32E-01
	NGTDM_Strength	0.01 ± 0.00	0.02 ± 0.02	3.28E-06	0.03 ± 0.09	0.02 ± 0.02	3.28E-01	0.02 ± 0.03	0.01 ± 0.01	2.47E-01
